# FBXO2-mediated KPTN ubiquitination promotes amino acid–dependent mTORC1 signaling and tumor growth

**DOI:** 10.1172/JCI195031

**Published:** 2025-12-16

**Authors:** Jianfang Gao, Jina Qing, Xianglong Li, Yuxuan Luo, Lingwen Huang, Hongxia Li, Huan Zhang, Jiao Zhang, Pei Xiao, Jinsong Li, Tingting Li, Shanping He

**Affiliations:** 1 Hunan Provincial Key Laboratory of Animal Intestinal Function and Regulation, Hunan International Joint Laboratory of Animal Intestinal Ecology and Health, Institute of Interdisciplinary Studies, College of Life Sciences, Hunan Normal University, Changsha, Hunan, China.; 2 Department of Spine Surgery, The Third Xiangya Hospital of Central South University, Changsha, Hunan, China.

**Keywords:** Cell biology, Oncology, Liver cancer, Signal transduction, Ubiquitin-proteosome system

## Abstract

Mechanistic target of rapamycin complex 1 (mTORC1) is a master controller of cell growth, and its dysregulation is associated with cancer. KICSTOR, a complex comprising KPTN, ITFG2, C12orf66, and SZT2, functions as a critical negative regulator of amino acid–induced mTORC1 activation. However, the regulatory mechanisms governing KICSTOR remain largely unclear. In this study, we identify F-box only protein 2 (FBXO2) as a key modulator of amino acid–dependent mTORC1 signaling. Mechanistically, FBXO2 colocalizes and directly interacts with KPTN via its F-box–associated domain, promoting K48- and K63-linked polyubiquitination of KPTN at lysine residues 49, 67, 262, and 265. FBXO2-mediated KPTN ubiquitination disrupted its interaction with ITFG2 and SZT2, while enhancing its interaction with C12orf66, thereby impairing the ability of KICSTOR to recruit the GATOR1 complex — comprising DEPDC5, NPRL2, and NPRL3 — to the lysosomal surface. Notably, FBXO2 protein levels were substantially upregulated in patients with liver cancer, and FBXO2-mediated KPTN ubiquitination facilitated the progression of hepatocellular carcinoma (HCC). These results reveal a key regulatory mechanism of mTORC1 signaling and highlight FBXO2 and KPTN ubiquitination as therapeutic targets for HCC treatment.

## Graphical abstract

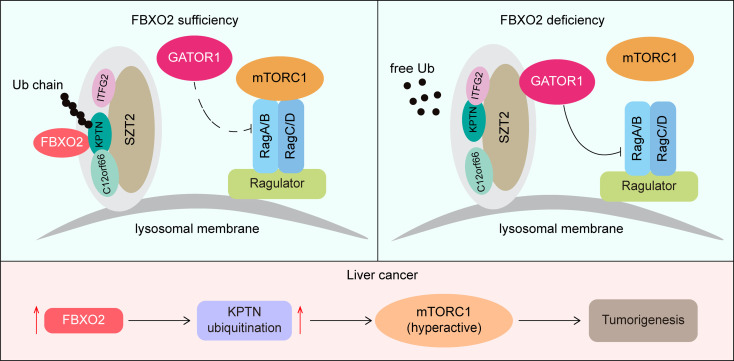

## Introduction

Mechanistic target of rapamycin complex 1 (mTORC1), composed of mTOR, Raptor, and mLST8, is a master controller of cell growth by integrating diverse signals, such as amino acids, glucose, lipids, and growth factors. As a kinase complex, mTORC1 governs anabolic and catabolic processes, including protein synthesis and autophagy, by phosphorylating key substrates, such as ribosomal protein S6 kinase 1 (S6K1), eukaryotic translation initiation factor 4E-binding protein 1 (4E-BP1), Unc-51-like kinase 1 (ULK1), and transcription factor EB (TFEB) (1–3). Dysregulation of the mTORC1 signaling pathway has been implicated in a variety of human diseases, including cancer, metabolic disorders, and neurodevelopmental disorders (2, 4, 5).

Amino acid–dependent activation of mTORC1 is mediated by heterodimeric Rag GTPases, consisting of RagA (or RagB) and RagC (or RagD) (6, 7). In response to amino acids, RagA/B are loaded with GTP and form active heterodimers with GDP-loaded RagC/D. These active Rag GTPases recruit mTORC1 to the lysosomal surface, where it is activated by the small GTPase Rheb (8–11). The nucleotide loading state of Rag GTPases is regulated by several protein complexes, including Ragulator, GATOR1, and FLCN-FNIP2 (4). Among these, the GATOR1 complex — comprising DEPDC5, NPRL2, and NPRL3 — acts as a GAP for RagA/B, thereby inhibiting amino acid–stimulated mTORC1 activation (12, 13). Notably, the lysosomal localization of GATOR1 is essential for its GAP function. KICSTOR, a lysosomal tethering complex composed of KPTN, ITFG2, C12orf66, and SZT2, anchors GATOR1 to the lysosome and thereby negatively regulates mTORC1 signaling (14, 15). Mutations of KPTN and SZT2 genes have been linked to neurodevelopmental disorders characterized by hyperactive mTORC1 signaling, highlighting the crucial role of KICSTOR in this pathway (14). Despite these findings, the potential existence of yet-unidentified regulators involved in amino acid–induced mTORC1 activation warrants further investigation to fully elucidate the complexities of this signaling network.

In this study, we identify F-box only protein 2 (FBXO2) as a key positive regulator of amino acid–dependent mTORC1 signaling. FBXO2 directly interacts with KPTN and promotes its K48- and K63-linked polyubiquitination at lysine residues 49, 67, 262, and 265. This posttranslational modification disrupts KICSTOR complex assembly and impairs the lysosomal recruitment of GATOR1. Bioinformatic analyses revealed that FBXO2 is upregulated in multiple cancer types, a finding further validated in human liver cancer tissues. Functionally, FBXO2-mediated KPTN ubiquitination promotes hepatocellular carcinoma (HCC) progression by enhancing mTORC1 activity both in vitro and in vivo. Collectively, these findings uncover a crucial regulatory mechanism within the mTORC1 pathway and highlight FBXO2 and KPTN ubiquitination as potential therapeutic targets for HCC.

## Results

### FBXO2 promotes amino acid–induced mTORC1 activation.

**Figure 1:**
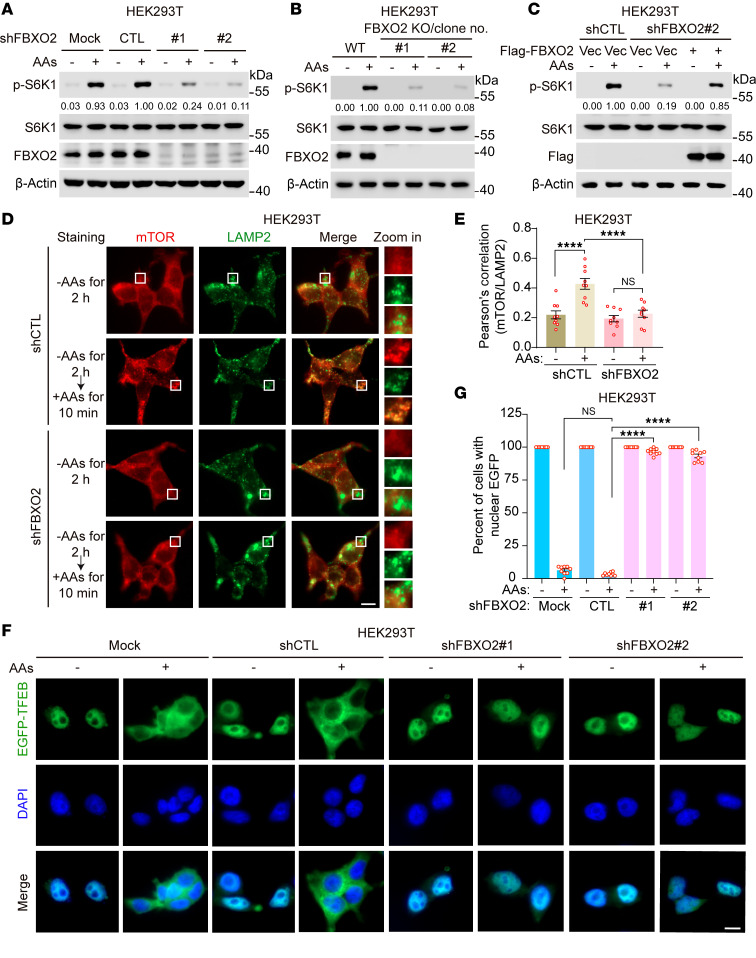
FBXO2 promotes amino acid–induced mTORC1 activation. (**A** and **B**) HEK293T cells stably expressing shRNAs against FBXO2 (shFBXO2) (**A**) or FBXO2-knockout cells (**B**) were deprived of amino acids and serum for 2 hours, then stimulated with amino acids for 10 minutes. Whole cell lysates (WCLs) were analyzed by immunoblotting with the indicated antibodies. AAs, amino acids. (**C**) HEK293T cells stably expressing shFBXO2 were transduced with lentiviruses expressing Flag-FBXO2-rsm (resistant to shFBXO2). WCLs of the cells treated as in (**A** and **B**) were analyzed by immunoblots. (**D** and **E**) HEK293T cells stably expressing the indicated shRNAs were deprived of amino acids and serum for 2 hours, then stimulated with amino acids for 10 minutes, followed by immunofluorescence analysis with the indicated antibodies. Representative images were shown (**D**), and colocalization of mTOR with LAMP2 was quantified (**E**). Scale bar, 10 μm. Insets were digitally zoomed. (**F** and **G**) HEK293T cells stably expressing EGFP-tagged TFEB (EGFP-TFEB) were transduced with lentiviruses expressing the indicated shRNAs for 72 hours. The transduced cells were deprived of amino acids and serum for 2 hours, then stimulated with amino acids for 2 hours, followed by nucleus staining with DAPI. Representative images of EGFP-TFEB localization were shown in **F**, and the quantitative results of EGFP-TFEB localization were presented in **G**. Scale bar, 10 μm. Data are presented as means ± SEM; *n* = 9 independent fields per condition; ****P < 0.0001, 1-way ANOVA followed by Tukey’s multiple comparisons test (**E** and **G**). Data are representative of at least 2 independent experiments (**A**–**C**).

To identify key regulators of mTORC1 activity, we utilized the interactome database BioGRID and identified 15 proteins potentially interacting with components of the amino acid–dependent mTORC1 signaling pathway (Supplemental Figure 1A; supplemental material available online with this article; https://doi.org/10.1172/JCI195031DS1) (16). We then employed 2 distinct short hairpin RNAs (shRNAs) to knock down each candidate gene and assessed their impacts on mTORC1 activity. Notably, FBXO2 knockdown resulted in the most robust inhibition of mTORC1 activity, as evidenced by an approximately 90% reduction in S6K1 phosphorylation at T389 (Supplemental Figure 1B). These findings were further validated in both HEK293T and HeLa cells (Figure 1A and Supplemental Figure 1C). Moreover, FBXO2 depletion via CRISPR/Cas9-mediated knockout substantially suppressed amino acid–stimulated mTORC1 activation (Figure 1B). Consistent with these results, FBXO2 transduction in FBXO2-depleted cells restored mTORC1 activity (Figure 1C). Amino acids, but not growth factors, activate mTORC1 by inducing the localization of mTOR to the lysosome, leading to the phosphorylation and subsequent cytoplasmic retardation of TFEB (17, 18). Silencing FBXO2 almost completely impaired amino acid–induced mTOR localization to lysosomes (Figure 1, D and E) and TFEB translocation from the nucleus to the cytoplasm (Figure 1, F and G, and Supplemental Figure 1, D and E). Additionally, mTORC1 plays a crucial role in suppressing autophagy (19). FBXO2 inhibition markedly enhanced basal autophagy, as indicated by increased LC3B-II levels (Supplemental Figure 1F) and the induction of EGFP-LC3B puncta (Supplemental Figure 1, G and H). Taken together, these results demonstrate that FBXO2 is essential for amino acid–induced mTORC1 activation.

### FBXO2 activates mTORC1 by directly binding to KPTN.

**Figure 2:**
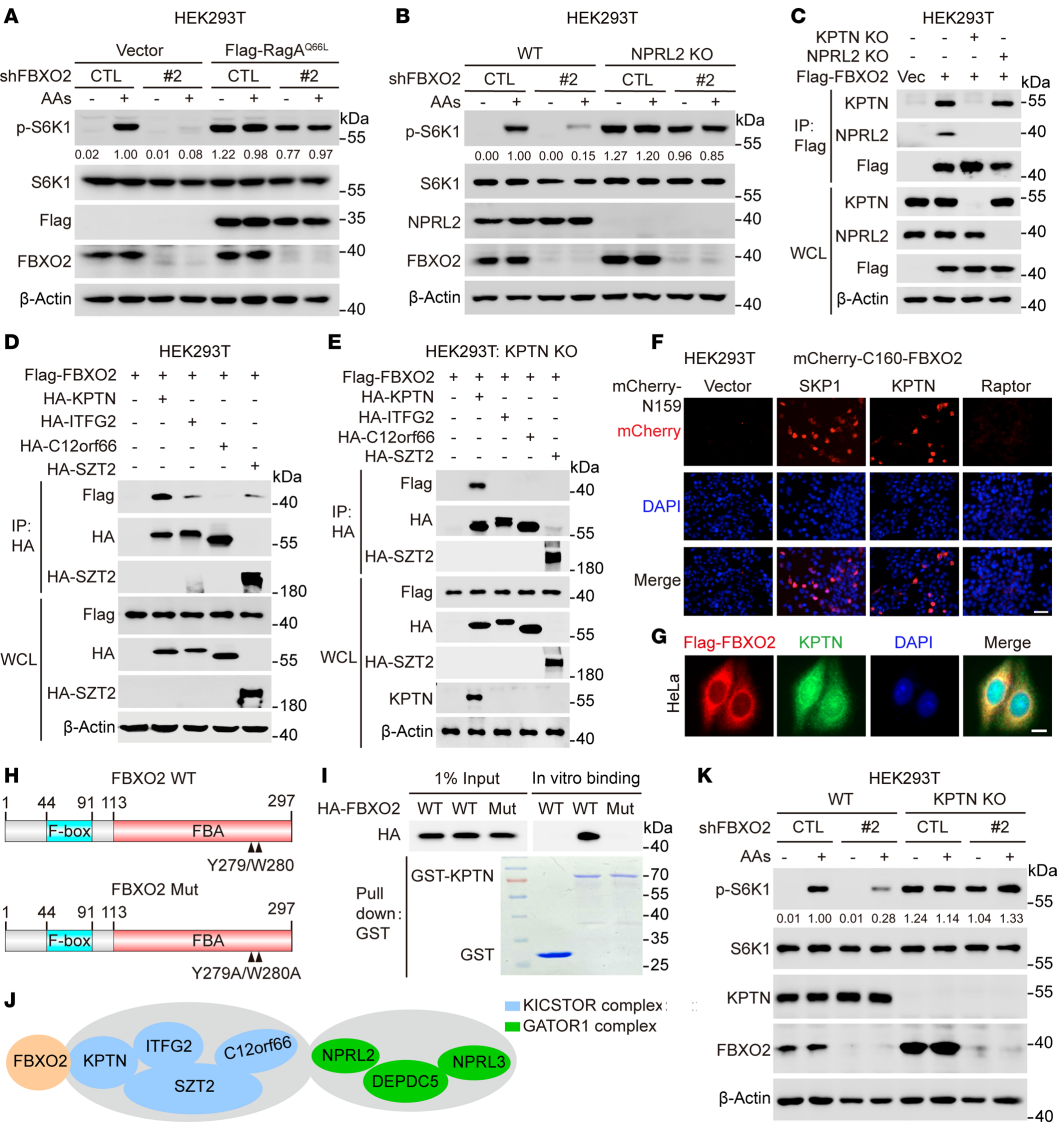
FBXO2 directly binds to KPTN via its F-box–associated domain. (**A** and **B**) HEK293T cells stably expressing Flag-tagged RagA-Q66L (Flag-RagA-Q66L) (**A**) or NPRL2-knockout cells (**B**) were transduced with lentiviruses expressing either shCTL or shFBXO2 for 72 hours, deprived of amino acids and serum for 2 hours, and then stimulated with amino acids for 10 minutes. WCLs were analyzed by immunoblotting with the indicated antibodies. (**C**) Wild-type (WT), KPTN-knockout, or NPRL2-knockout HEK293T cells were transduced with lentiviruses expressing Flag-FBXO2. (**D** and **E**) HEK293T cells stably expressing Flag-FBXO2 (**D**) or KPTN-knockout HEK293T cells (**E**) were transfected with plasmids containing the indicated genes. WCLs (**C**–**E**) were immunoprecipitated with anti-Flag (**C**) or anti-HA (**D** and **E**) magnetic beads, followed by immunoblotting with the indicated antibodies. (**F**) HEK293T cells were cotransfected with plasmids expressing mCherry-C160–tagged FBXO2 (mCherry-C160-FBXO2) and mCherry-N159–tagged SKP1, KPTN, or Raptor, followed by nucleus staining with DAPI. Representative images of mCherry were shown. Scale bar, 100 μm. (**G**) HeLa cells stably expressing Flag-FBXO2 were subjected to immunofluorescence assays with the indicated antibodies. Scale bar, 10 μm. (**H**) Schematic diagram of the domains of FBXO2. FBA, F-box–associated. (**I**) WCLs of HEK293T cells transfected with plasmids expressing HA-tagged FBXO2 or its mutant FBXO2-Y279A/W280A were incubated with purified GST-tagged KPTN (GST-KPTN) or GST proteins from bacteria, followed by in vitro GST pulldown assays. GST, glutathione-S-transferase. (**J**) Diagram of FBXO2 interacting with KPTN rather than other components of KICSTOR or GATOR1. (**K**) WT or KPTN-knockout HEK293T cells were transduced with lentiviruses expressing either shCTL or shFBXO2 for 72 hours, deprived of amino acids and serum for 2 hours, and then stimulated with amino acids for 10 minutes. WCLs were analyzed by immunoblotting with the indicated antibodies. Data are representative of at least 2 independent experiments (**A**–**E**, **I**, and **K**).

To explore how FBXO2 regulates mTORC1 activity, we conducted epistatic analyses between FBXO2 and key components of amino acid–dependent mTORC1 signaling, including RagA and NPRL2. Consistent with previous reports, overexpression of active RagA mutant (RagA-Q66L) or NPRL2 depletion rendered cells resistant to amino acid starvation, leading to constitutively activated mTORC1 (Figure 2, A and B) (6, 13). FBXO2 knockdown substantially suppressed amino acid–induced mTORC1 activation in control cells, but not in RagA-Q66L–expressing or NPRL2-knockout cells, indicating that FBXO2 acts upstream of both GATOR1 and Rag GTPases to regulate mTORC1 (Figure 2, A and B).

Co-immunoprecipitation (co-IP) assays revealed that FBXO2 strongly associated with KPTN, weakly bound to NPRL2, and barely interacted with mTORC1 and RagA/C complexes (Supplemental Figure 2A). KPTN depletion disrupted the FBXO2-NPRL2 interaction, whereas NPRL2 knockout did not affect FBXO2’s association with KPTN, indicating that KPTN mediates the FBXO2-NPRL2 interaction (Figure 2C). To assess the specificity of the FBXO2-KPTN interaction, we included a related F-box protein, FBXO6, as a control. Notably, FBXO2, but not FBXO6, bound to KPTN, and this interaction was largely insensitive to amino acid stimulation (Supplemental Figure 2, B and C).

Given that KPTN is a key component of KICSTOR complex, we next examined whether FBXO2 interacts with other components of this complex, including C12orf66, ITFG2, and SZT2. As expected, FBXO2 readily interacted with KPTN, whereas it weakly bound with ITFG2 and SZT2, both of which are direct binding partners of KPTN (Figure 2D). In contrast, no detectable interaction between FBXO2 and C12orf66 was observed under the experimental conditions, likely due to the indirect association between KPTN and C12orf66 (14) (Figure 2D). Notably, knockout of KPTN completely abolished the interactions of FBXO2 with ITFG2 and SZT2, indicating that FBXO2’s association with the KICSTOR complex is mediated through KPTN (Figure 2E). Furthermore, bimolecular fluorescence complementation (BiFC) assays demonstrated that FBXO2 associated with KPTN, but not with Raptor, to a comparable extent as with SKP1, a well-characterized FBXO2 binding partner (Figure 2F). In addition, FBXO2 colocalized with KPTN in the cytoplasm (Figure 2G). Structurally, FBXO2 contains an N-terminal F-box domain and a C-terminal F-box–associated (FBA) domain, which is responsible for substrate recognition (Figure 2H). Previous reports have shown that mutations of key residues Y279 and W280 within the FBA domain (FBXO2-Mut) impair FBXO2’s ability to bind substrates (20). Consistent with this, GST pulldown assays revealed that WT FBXO2 bound KPTN, while FBXO2-Mut did not (Figure 2I). These results indicate that FBXO2 interacts with KICSTOR and GATOR1 complexes by directly binding to KPTN via its FBA domain and that this interaction is not regulated by amino acid availability (Figure 2J).

We next investigated the functional consequences of FBXO2’s interaction with the KICSTOR complex. Consistently, FBXO2 knockdown markedly suppressed amino acid–induced mTORC1 activation, an effect that was substantially reversed by KPTN depletion (Figure 2K). These findings suggest that FBXO2 promotes mTORC1 activation through its interaction with KPTN.

### FBXO2 activates mTORC1 by promoting KPTN ubiquitination.

**Figure 3:**
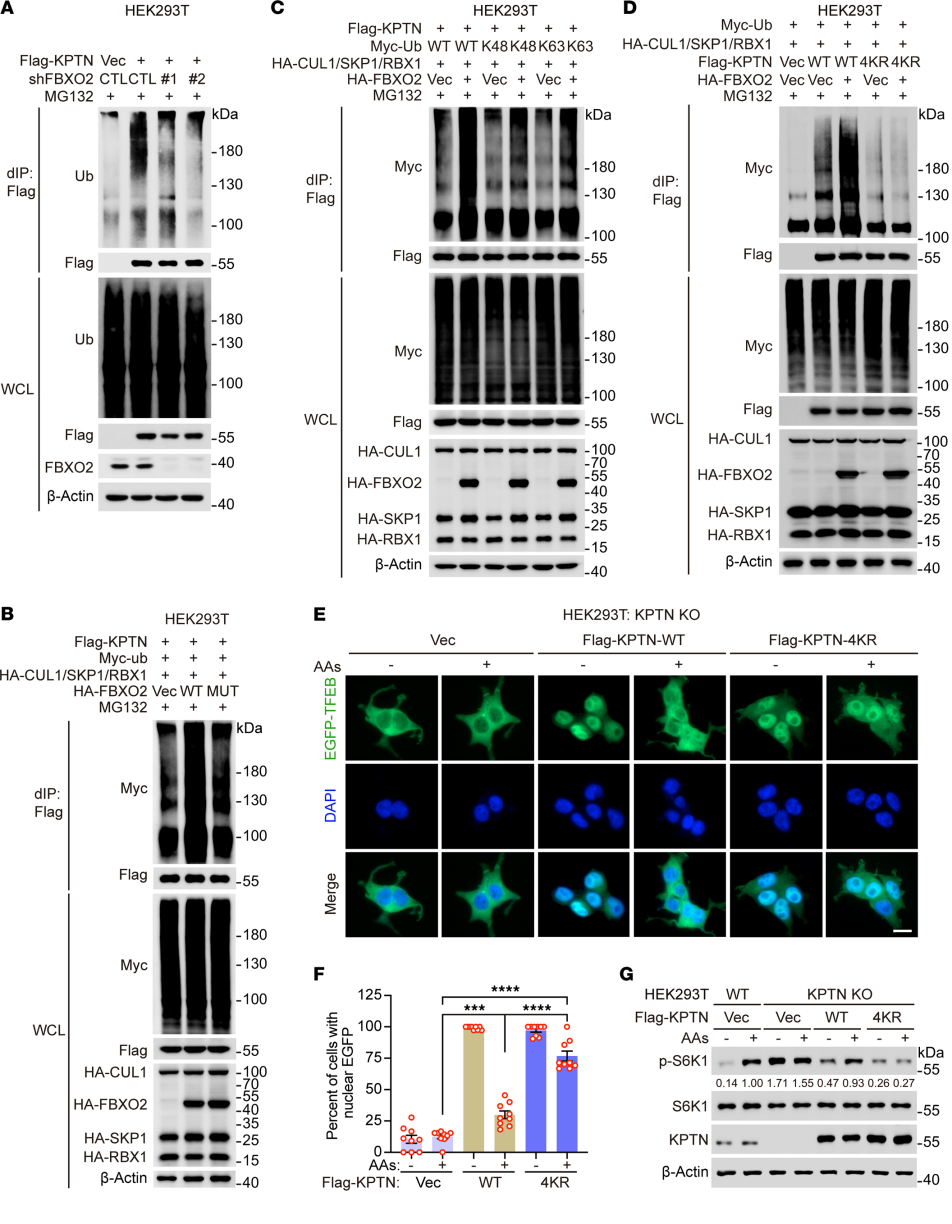
FBXO2 activates mTORC1 by promoting KPTN ubiquitination. (**A** and **B**) HEK293T cells stably expressing Flag-KPTN were transduced with lentiviruses expressing the indicated shRNAs (**A**) or transfected with plasmids expressing the indicated genes (**B**), then treated with MG132 (20 μM) for 12 hours at 60 hours posttransduction or 36 hours posttransfection. (**C** and **D**) HEK293T cells stably expressing Flag-KPTN (**C**) or HEK293T cells (**D**) were transfected with plasmids expressing the indicated genes, then treated with MG132 (20 μM) for 12 hours at 60 hours posttransfection. WT, wild-type; Ub-K48, with the other 6 lysine residues of ubiquitin (Ub) mutated to arginine except lysine 48 (K48); Ub-K63, with the other 6 lysine residues of Ub mutated to arginine except lysine 63 (K63); KPTN-4KR, KPTN-K49/67/262/265R. WCLs (**A**–**D**) were denatured and then immunoprecipitated with anti-Flag magnetic beads, followed by immunoblotting with the indicated antibodies. (**E** and **F**) KPTN-knockout HEK293T cells stably expressing EGFP-TFEB were transduced with lentiviruses expressing Flag-tagged KPTN or KPTN-4KR for 24 hours, deprived of amino acids and serum for 2 hours, and stimulated with amino acids for 2 hours, followed by nucleus staining with DAPI. Representative images of EGFP-TFEB localization were shown (**E**), and the quantitative results of EGFP-TFEB localization were presented (**F**). Scale bar, 10 μm. Data are presented as means ± SEM; *n* = 9 independent fields per condition; ***P < 0.001, ****P < 0.0001, 1-way ANOVA followed by Tukey’s multiple comparisons test. (**G**) WT and KPTN-knockout HEK293T cells were transduced with lentiviruses expressing Flag-tagged KPTN or KPTN-4KR for 24 hours, deprived of amino acids and serum for 2 hours, and stimulated with amino acids for 10 minutes. WCLs were analyzed by immunoblotting with the indicated antibodies. Data are representative of at least 2 independent experiments (**A**–**D** and **G**). dIP, denaturing immunoprecipitation.

FBXO2 is a subunit of the SKP1-CUL1-F-box protein (SCF) E3 ubiquitin-protein ligase complex, which mediates the ubiquitination of target proteins. As expected, knockdown of FBXO2 substantially decreased the levels of KPTN ubiquitination, whereas overexpression of WT FBXO2, but not its mutant form incapable of interacting with KPTN, markedly increased KPTN polyubiquitination (Figure 3, A and B). FBXO6, which did not interact with KPTN, failed to enhance KPTN ubiquitination (Supplemental Figure 3A).

We next tested whether nutrient status regulates FBXO2 activity. FBXO2 directly binds SKP1 within the SCF complex (21), and this interaction was unaffected by amino acid stimulation, indicating that SCF^FBXO2^ assembly is nutrient independent (Supplemental Figure 3B). Moreover, FBXO2 protein abundance and subcellular localization remained unchanged upon amino acid stimulation (Supplemental Figure 3, C–E). Database analysis using PhosphoSitePlus (https://www.phosphosite.org/homeAction.action) revealed multiple putative phosphorylation sites rather than other types of posttranslational modifications in FBXO2; however, only negligible changes in FBXO2 phosphorylation were observed after amino acid stimulation (Supplemental Figure 3F). Consistent with these findings, FBXO2-mediated KPTN ubiquitination was insensitive to nutrient availability (Supplemental Figure 3G). Together, these data indicate that FBXO2 constitutively promotes KPTN ubiquitination independent of amino acid status.

Using both WT Ub and linkage-specific mutants, we found that KPTN underwent K48- and K63-linked polyubiquitination, both of which were enhanced by FBXO2 overexpression (Supplemental Figure 4, A and B, and Figure 3C). To identify the specific ubiquitination sites on KPTN targeted by FBXO2, we screened 7 potential sites identified by mass spectrometry or predicted by bioinformatics analysis using GPS-Uber (Supplemental Figure 4C) (22, 23). Among these, lysine residues K49, K67, K262, and K265 were confirmed as the primary ubiquitination sites, as demonstrated by reduced ubiquitination in the KPTN-4KR mutant (KPTN-K49/67/262/265R) (Supplemental Figure 4D). Notably, the ubiquitination level of KPTN-4KR was severely diminished and comparable to that of KPTN-K0, in which all lysine residues were substituted with arginine (Supplemental Figure 4D). While WT KPTN exhibited increased ubiquitination upon FBXO2 overexpression, the KPTN-4KR mutant did not, though it retained the ability to associate with FBXO2 (Figure 3D and Supplemental Figure 4E). These results indicate that FBXO2 promotes K48- and K63-linked polyubiquitination of KPTN at K49, K67, K262, and K265.

Next, we examined the functional consequence of FBXO2-mediated KPTN ubiquitination in regulating mTORC1 activity. Reexpression of KPTN substantially inhibited amino acid–induced mTORC1 activation in KPTN-depleted cells, as evidenced by reduced p-S6K levels and increased retention of TFEB in the nucleus (Figure 3, E–G). Notably, these inhibitory effects were more pronounced following expression of the KPTN-4KR mutant compared with WT KPTN (Figure 3, E–G). Taken together, these results indicate that FBXO2 activates mTORC1 by mediating KPTN ubiquitination at multiple lysine residues.

### FBXO2-mediated KPTN ubiquitination suppresses KICSTOR recruitment of GATOR1.

We further investigated how FBXO2-mediated KPTN ubiquitination regulates mTORC1 activity. As ubiquitination commonly affects protein stability and protein-protein interactions, we first examined whether FBXO2 influences KPTN protein levels. FBXO2 knockdown had minimal effect on KPTN expression (Supplemental Figure 5A), and the protein levels of the KPTN-4KR mutant were comparable to those of WT KPTN (Supplemental Figure 5B). These results indicate that FBXO2-mediated KPTN polyubiquitination does not regulate its protein stability.

**Figure 4:**
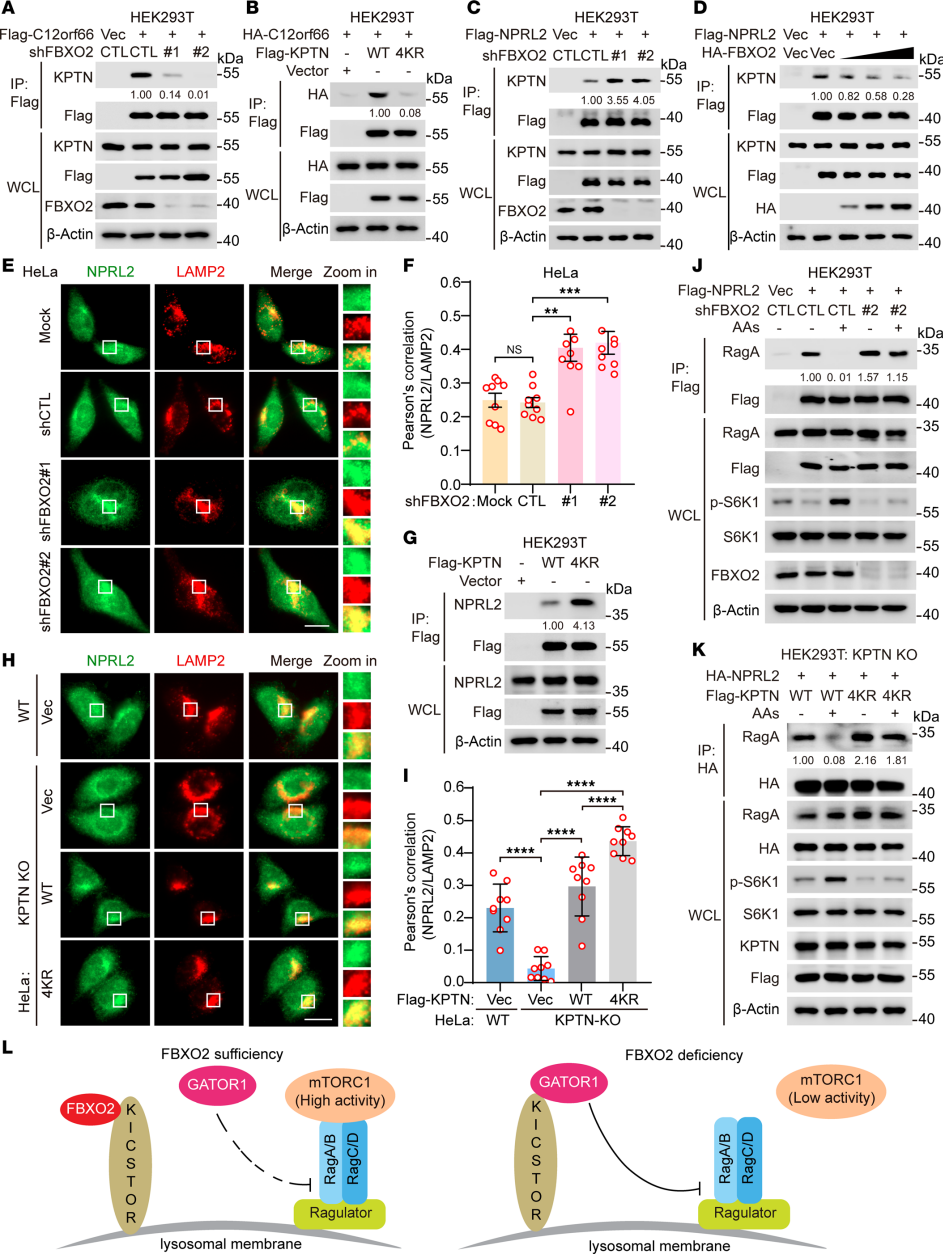
FBXO2-mediated KPTN ubiquitination suppresses KICSTOR functions. (**A**–**D**) HEK293T cells stably expressing Flag-C12orf66 (**A**), HA-C12orf66 (**B**), or Flag-NPRL2 (**C** and **D**) were transduced with lentiviruses expressing the indicated shRNAs or genes (**A**–**C**) or transfected with increasing amounts of plasmids expressing HA-FBXO2 (**D**). WCLs (**A**–**D**) were immunoprecipitated with anti-Flag magnetic beads, followed by immunoblotting with the indicated antibodies. (**E** and **F**) HeLa cells transduced with lentiviruses expressing shFBXO2 were subjected to immunofluorescence assays with the indicated antibodies. Representative images were shown (**E**), and colocalization of NPRL2 with LAMP2 was quantified (**F**). Scale bar, 10 μm. Insets were digitally zoomed.(**G**) HEK293T cells were transduced with lentiviruses expressing the indicated genes. WCLs were immunoprecipitated with anti-Flag magnetic beads, followed by immunoblotting with the indicated antibodies. (**H** and **I**) KPTN-knockout HeLa cells transduced with lentiviruses expressing the indicated genes were subjected to immunofluorescence assays with the indicated antibodies. Representative images were shown (**H**), and colocalization of NPRL2 with LAMP2 was quantified (**I**). Scale bar, 10 μm. Insets were digitally zoomed. (**J** and **K**) HEK293T cells stably expressing Flag-NPRL2 (**J**) or KPTN-knockout HEK293T cells stably expressing HA-NPRL2 (**K**) were transduced with lentiviruses expressing the indicated shRNAs or genes. The transduced cells were deprived of amino acids and serum for 2 hours, then stimulated with amino acids for 10 minutes. WCLs were immunoprecipitated with anti-Flag (**J**) or anti-HA (**K**) magnetic beads, followed by immunoblotting with the indicated antibodies. (**L**) A model depicting how FBXO2 regulates the GATOR1 lysosome localization and mTORC1 activity. Data are presented as means ± SEM; *n* = 9 independent fields per condition; **P < 0.01, ***P < 0.001, ****P < 0.0001, 1-way ANOVA followed by Tukey’s multiple comparisons test (**F** and **I**). Data are representative of at least 2 independent experiments (**A**–**D**, **G**, **J**, and **K**).

Next, we assessed whether FBXO2-mediated ubiquitination modulates the interaction of KPTN with other KICSTOR components. Notably, FBXO2 inhibition markedly reduced the association between KPTN and C12orf66 (Figure 4A). Intriguingly, FBXO2 knockdown enhanced, whereas FBXO2 overexpression inhibited, the interaction of KPTN with ITFG2 (Supplemental Figure 5, C and D). In contrast, the ITFG2-C12orf66 interaction remained unchanged following FBXO2 knockdown, while the association between KPTN and SZT2 was modestly increased (Supplemental Figure 5, E and F). In agreement with these observations, the KPTN-4KR mutant, which lacks the 4 ubiquitination sites targeted by FBXO2, exhibited a stronger affinity for both ITFG2 and SZT2 and a weaker interaction with C12orf66 compared with WT KPTN (Supplemental Figure 5, G–I, and Figure 4B). Collectively, these results indicate that FBXO2-mediated KPTN ubiquitination disrupts KICSTOR complex assembly by suppressing KPTN interaction with both ITFG2 and SZT2 while promoting its association with C12orf66.

Given that the KICSTOR complex functions as an anchor for the GATOR1 complex at the lysosomal surface (14), we next examined whether FBXO2-mediated KPTN ubiquitination affects the KICSTOR-GATOR1 interaction. FBXO2 knockdown enhanced the interaction between KPTN and NPRL2, a key component of the GATOR1 complex, while FBXO2 overexpression reduced this interaction in a dose-dependent manner (Figure 4, C and D). Consequently, depletion of FBXO2 enhanced the lysosomal localization of NPRL2, as evidenced by the increased colocalization of NPRL2 with LAMP2 (Figure 4, E and F). Consistent with these results, the interaction of NPRL2 with KPTN-4KR was stronger than that with WT KPTN (Figure 4G). Accordingly, KPTN-4KR overexpression induced greater colocalization of NPRL2 with LAMP2 compared with WT KPTN, suggesting that FBXO2-mediated KPTN ubiquitination impairs the lysosomal localization of GATOR1 (Figure 4, H and I).

Since KICSTOR is essential for GATOR1 interaction with RagA at the lysosome in response to amino acid deprivation (14), we next assessed whether FBXO2-mediated KPTN ubiquitination influences the GATOR1-RagA interaction. Whereas amino acid stimulation abolished NPRL2-RagA interaction, silencing FBXO2 resulted in a constitutive association of NPRL2 with RagA regardless of amino acid availability (Figure 4J). Accordingly, the interaction of RagA with KPTN-4KR mutant was stronger than that with WT KPTN in both the absence and presence of amino acids (Figure 4K). Together, these results demonstrate that FBXO2-mediated KPTN ubiquitination suppresses the lysosomal localization of GATOR1 and subsequent GATOR1-RagA interaction (Figure 4L).

### FBXO2 promotes the progression of HCC by activating mTORC1.

**Figure 5:**
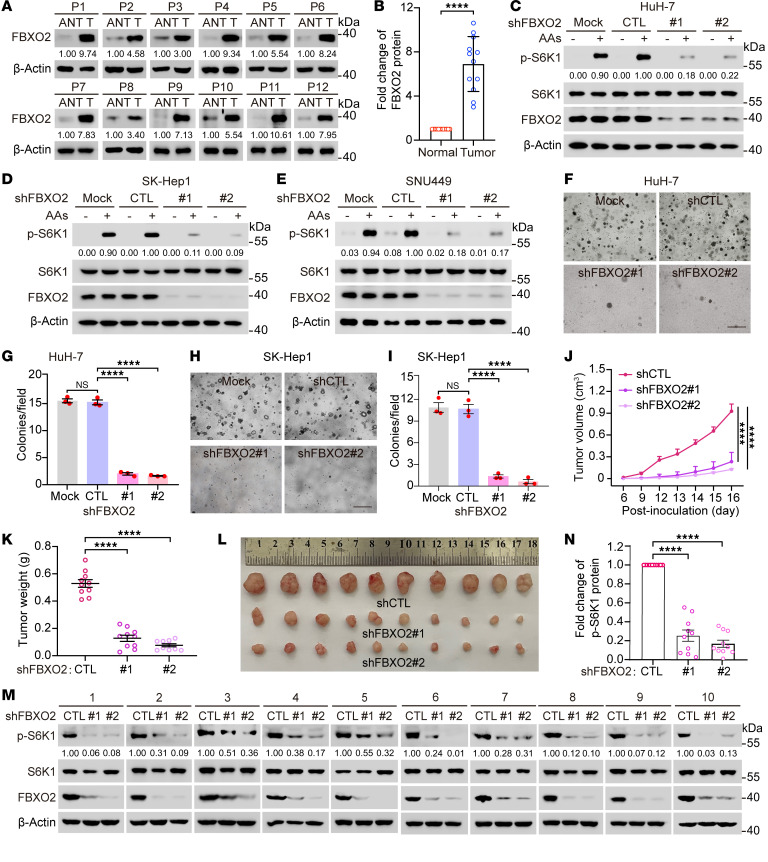
FBXO2 promotes the progression of HCC by activating mTORC1. (**A**) Liver tumors (T) and paired adjacent normal tissues (ANT) from patients were analyzed by immunoblotting with the indicated antibodies. (**B**) The relative protein levels of FBXO2 (**A**) were quantified. Data are presented as means ± SEM; *n* = 12; ****P < 0.0001, unpaired 2-tailed Student’s *t* test. (**C**–**E**) HuH-7 (**C**), SK-Hep1 (**D**), and SNU449 (**E**) cells stably expressing shFBXO2 were deprived of amino acids and serum for 2–3 hours, then stimulated with amino acids for 20 minutes. WCLs were analyzed by immunoblotting with the indicated antibodies. (**F**–**I**) HuH-7 (**F** and **G**) and SK-Hep1 (**H** and **I**) cells were transduced with lentiviruses expressing shCTL or shFBXO2 for 24 hours and then seeded in 6-well plates to be examined for colony formation in soft agar. Representative pictures at 4× objective were shown (**F** and **H**), and colonies with diameter > 10 μm were quantified (**G** and **I**). Scale bar, 100 μm. Data are presented as means ± SEM; *n* = 3 biologically independent repeats; ****P < 0.0001, 1-way ANOVA followed by Tukey’s multiple comparisons test. (**J**–**L**) HuH-7 cells stably expressing shCTL or shFBXO2 were subcutaneously injected into nude mice for xenograft growth. The tumor volumes at the indicated days postinoculation (**J**) and the tumor weights of the last time point (**K**) were measured, and the tumors of the last time point were photographed (**L**). (**M**) The xenograft tumors (**L**) were analyzed by immunoblotting with the indicated antibodies. (**N**) The relative levels of p-S6K1 (**M**) were quantified. Data are presented as means ± SEM; *n* = 10; ****P < 0.0001, 1-way ANOVA followed by Tukey’s multiple comparisons test (**J**, **K**, and **N**). Data are representative of at least 2 independent experiments (**C**–**E**).

Dysregulation of mTORC1 is closely associated with tumorigenesis, and mTORC1 hyperactivation has been reported in up to 80% of human cancers (4, 24). Analysis of the TNMplot database revealed that FBXO2 was dramatically upregulated in multiple cancer types, including liver, ovary, thyroid, and uterus cancers (25) (Supplemental Figure 6A). Since the oncogenic roles of FBXO2 in ovarian, thyroid, and uterine cancers have been investigated (26–28), we focused on liver cancer to explore its function in a pathological context that remains less well characterized. Consistent with the TNMplot data, the protein levels of FBXO2 were markedly elevated in human liver cancer tissues compared with adjacent normal tissues (Figure 5, A and B). Moreover, FBXO2 knockdown notably inhibited amino acid–induced mTORC1 activation in several HCC cell lines, including HuH-7, SK-Hep1, and SNU449 (Figure 5, C–E). In addition, FBXO2 depletion suppressed HCC cell proliferation, colony formation, and anchorage-independent growth (Supplemental Figure 6, B–H, and Figure 5, F–I). To evaluate the role of FBXO2 in HCC development in vivo, we subcutaneously transplanted FBXO2-depleted or control HuH-7 cells into nude mice. Knockdown of FBXO2 substantially suppressed the xenograft tumor growth and mTORC1 activity in the tumors (Figure 5, J–N). Together, these results indicate that FBXO2 promotes the progression of HCC by activating mTORC1 both in vitro and in vivo.

### FBXO2-mediated KPTN ubiquitination drives HCC progression via mTORC1 activation.

**Figure 6:**
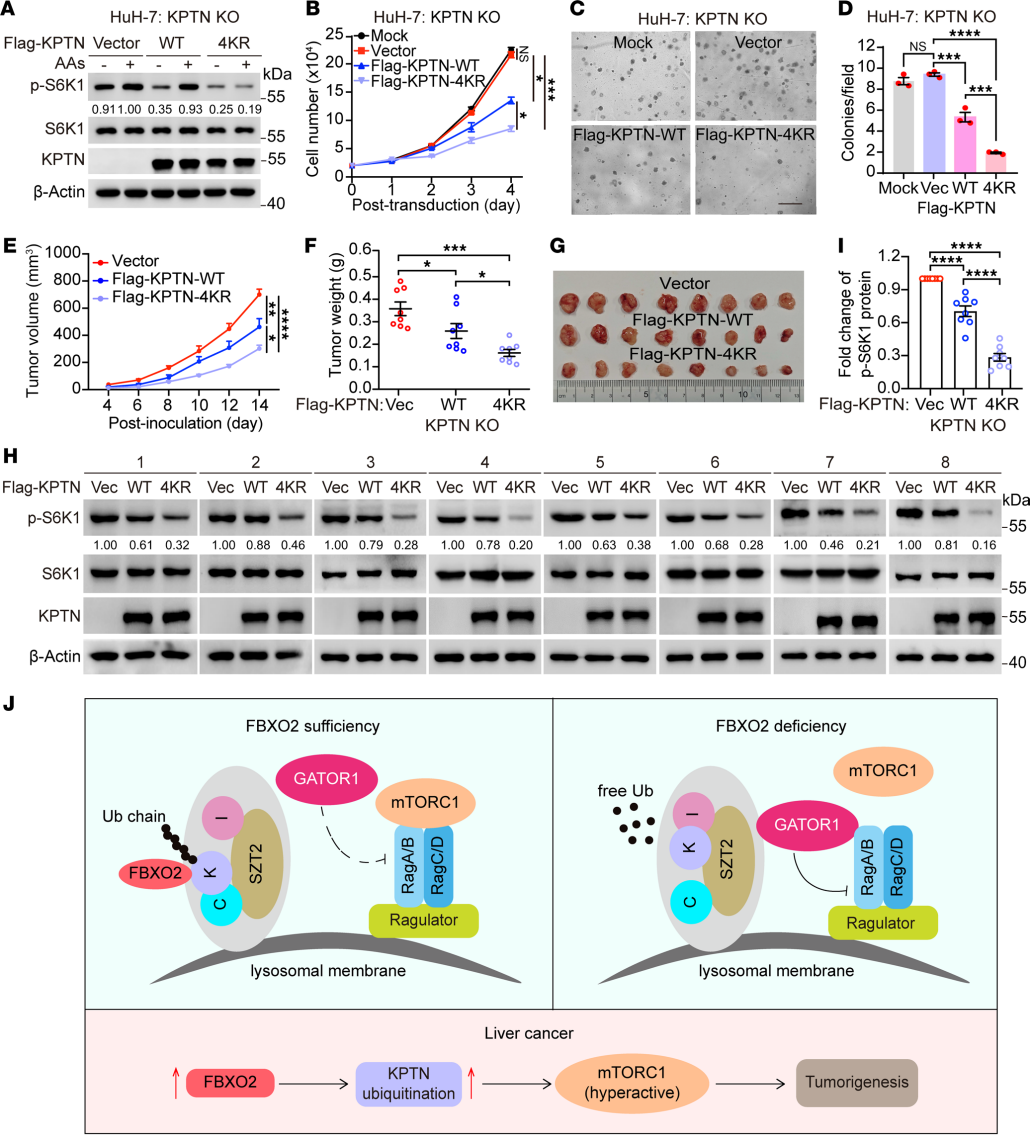
FBXO2-mediated KPTN ubiquitination drives HCC progression via mTORC1 activation. (**A**) KPTN-knockout HuH-7 cells were transduced with lentiviruses expressing WT KPTN or KPTN-4KR mutant, deprived of amino acids and serum for 2 hours, and stimulated with amino acids for 20 minutes. WCLs were analyzed by immunoblotting with the indicated antibodies. Data are representative of at least 2 independent experiments. (**B**–**D**) The transduced cells (**A**) were seeded in 24-well pates for cell proliferation (**B**) or in 6-well pates to be examined for colony formation in soft agar (**C** and **D**). Cell numbers were counted for 4 consecutive days (**B**). Representative pictures at 4× objective were shown (**C**), and colonies with diameter > 10 μm were quantified (**D**). Scale bar, 100 μm. Data are presented as means ± SEM; *n* = 3 biologically independent repeats; *P < 0.05, ***P < 0.001, ****P < 0.0001, 1-way ANOVA followed by Tukey’s multiple comparisons test. (**E**–**G**) The transduced cells in **A** were subcutaneously injected into nude mice for xenograft growth. The tumor volumes at the indicated days postinoculation (**E**) and the tumor weights of the last time point (**F**) were measured, and the tumors of the last time point were photographed (**G**). Data are presented as means ± SEM, *n* = 8; *P < 0.05, **P < 0.01, ***P < 0.001, ****P < 0.0001, 1-way ANOVA followed by Tukey’s multiple comparisons test. (**H**) The xenograft tumors (**G**) were analyzed by immunoblotting with the indicated antibodies. (**I**) The relative levels of p-S6K1 (**H**) were quantified. Data are presented as means ± SEM, *n* = 8; ****P < 0.0001, 1-way ANOVA followed by Tukey’s multiple comparisons test. (**J**) A model depicting how FBXO2-mediated KPTN ubiquitination promotes amino acid–dependent mTORC1 signaling and tumor growth.

To investigate the role of FBXO2-mediated KPTN ubiquitination in HCC progression, we first generated KPTN-knockout HuH-7 cells and reexpressed either WT KPTN or the ubiquitination-deficient KPTN-4KR mutant in these cells. Reexpression of WT KPTN substantially inhibited mTORC1 activity only under amino acid–deprived conditions (Figure 6A). Notably, the KPTN-4KR mutant, but not WT KPTN, abolished mTORC1 activation regardless of amino acid status (Figure 6A). These results indicate that KPTN-4KR suppresses mTORC1 activation more potently than WT KPTN. Consistent with these observations, KPTN 4KR more effectively inhibited the proliferation and anchorage-independent growth of KPTN-deficient HuH-7 cells than WT KPTN (Figure 6, B–D). In accord, KPTN-4KR more robustly suppressed xenograft tumor growth (Figure 6, E–G) and reduced mTORC1 activity in the tumors than WT KPTN (Figure 6, H and I). Collectively, these results indicate that FBXO2-mediated KPTN ubiquitination promotes the progression of HCC by activating mTORC1 both in vitro and in vivo (Figure 6J).

## Discussion

KICSTOR plays a critical role in amino acid sensing upstream of mTORC1 by recruiting GATOR1 to the lysosomal surface (14). In this study, we uncovered a crucial mechanism whereby FBXO2-mediated ubiquitination of KPTN regulates amino acid–dependent mTORC1 signaling through modulation of the KICSTOR complex (Figure 6J). FBXO2 directly interacts with KPTN to promote its K48- and K63-linked polyubiquitination, which in turn disrupts KPTN’s interactions with ITFG2 and SZT2 while enhancing its association with C12orf66. As a consequence, this ubiquitination impairs the lysosomal localization of GATOR1 and its interaction with RagA, thereby facilitating mTORC1 activation at the lysosomal membrane. Notably, FBXO2 expression is elevated in HCC, and FBXO2-mediated KPTN ubiquitination promotes HCC progression both in vitro and in vivo (Figure 6J).

Previous studies, including our own, have demonstrated that GATOR1 is subject to regulation by various posttranslational modifications, including ubiquitination, phosphorylation, and methylation (29–33). Recent reports have also identified VWCE and OTUD3 as KICSTOR regulators. OTUD3 interacts with KICSTOR, deubiquitinates KPTN, and promotes the lysosomal recruitment of GATOR1 through an as-yet-undefined mechanism (34). VWCE binds to KICSTOR and enhances the KPTN-C12orf66 interaction, thereby facilitating GATOR1 lysosomal localization (35). However, the precise mechanism by which VWCE regulates this interaction remains unclear. Distinct from these factors, we identified FBXO2 as a crucial KICSTOR regulator that directly binds to KPTN and indirectly associates with other KICSTOR components via KPTN. We propose that FBXO2-mediated ubiquitination of KPTN modulates the intracomplex balance of interactions, thereby influencing the structural integrity and functional activity of KICSTOR. It is reported that KPTN typically forms a heterodimer with ITFG2 within KICSTOR (14). Our data demonstrate that FBXO2-mediated ubiquitination of KPTN disrupts its interactions with ITFG2 and SZT2 while promoting its interaction with C12orf66, ultimately altering KICSTOR assembly and function. In contrast, VWCE modulates the KPTN-C12orf66 interaction without affecting KPTN-ITFG2 binding (35). Furthermore, FBXO2-mediated KPTN ubiquitination inhibits both GATOR1 lysosomal localization and its association with RagA, indicating a functional disruption of the KICSTOR complex. Structural characterization of KICSTOR in complex with FBXO2 will be essential to elucidate the precise molecular basis of this regulation. Given that KICSTOR constitutively anchors GATOR1 to lysosomes independent of amino acid availability (14), our findings show that FBXO2 regulates KICSTOR assembly and GATOR1 lysosomal recruitment through KPTN ubiquitination in an amino acid–insensitive manner. Similarly, VWCE has also been shown to modulate KICSTOR-GATOR1 association regardless of amino acid status (35).

Protein ubiquitination is increasingly recognized as a key regulator of amino acid–dependent mTORC1 signaling by modulating protein-protein interactions. For example, RNF167 and STAMBPL1 control K63-linked ubiquitination of Sestrin2 at lysine 175, modulating its interaction with GATOR2 (36). Likewise, RNF152 and SKP2 mediate K63-linked ubiquitination of RagA to enhance its binding to GATOR1, while WDR24 facilitates K6-linked ubiquitination of NPRL2 to inhibit its interaction with RagA (31, 37, 38). TRAF4 similarly promotes K63-linked ubiquitination of LAMTOR1 to facilitate its association with Rag GTPases (39). In line with these findings, FBXO2-mediated K48- and K63-linked polyubiquitination of KPTN at lysine residues 49, 67, 262, and 265 disrupts its interactions with ITFG2 and SZT2 while promoting its interaction with C12orf66, without altering KPTN protein levels. This modulation leads to impaired KICSTOR assembly and diminished GATOR1 lysosomal recruitment.

FBXO2 has previously been implicated as an oncogene in several cancers, including ovarian cancer (OV), endometrial carcinoma (EC), osteosarcoma (OS), and papillary thyroid carcinoma (PTC). It has been shown to promote the ubiquitination and degradation of FBN1, glycosylated SUN2, and p53 in EC, OV, and PTC, respectively (26–28). In contrast, in OS cells, FBXO2 stabilizes interleukin-6 receptor, thereby activating STAT3 signaling (40). In this study, we demonstrated that FBXO2 similarly acts as an oncogene in HCC by promoting amino acid–dependent mTORC1 activation. FBXO2 expression was markedly elevated in liver cancer, and through both FBXO2 knockdown and overexpression of a ubiquitination-deficient KPTN-4KR mutant, we established that FBXO2-mediated KPTN ubiquitination enhances amino acid–induced mTORC1 activation in HCC. This, in turn, promotes HCC cell proliferation, colony formation, anchorage-independent growth, and xenograft tumor growth. Unlike its oncogenic roles in other cancers, where FBXO2 primarily regulates protein stability (26–28, 40), in HCC, FBXO2-mediated KPTN ubiquitination modulates protein-protein interactions within KICSTOR without affecting KPTN protein levels.

Beyond cancer, FBXO2 is also involved in regulating essential physiological processes such as glucose homeostasis, mitophagy, and ferroptosis (41, 42). Therefore, broad systemic inhibition of FBXO2 function may cause unwanted toxicity by perturbing normal cellular functions. To mitigate this concern, our findings suggest that more selective strategies, such as specifically disrupting the FBXO2-KPTN interaction, for example through inhibitory peptides, could provide a safer and more targeted therapeutic approach for treating mTORC1-hyperactivated HCC.

In conclusion, this study reveals a crucial mechanism by which FBXO2-mediated ubiquitination of KPTN modulates amino acid–dependent mTORC1 signaling and drives HCC progression through regulation of the KICSTOR complex. These findings advance our understanding of mTORC1 regulation and suggest that both FBXO2 and KPTN ubiquitination represent promising therapeutic targets for HCC.

## Methods

### Sex as a biological variable.

Our animal experiments used animals of a single sex (male animals for Figure 5, J–N, and female animals for Figure 6, E–I) because sex is considered a biological variable that can influence subcutaneous xenograft tumor growth. The findings are expected to be relevant to both sexes.

### Antibodies.

Antibodies against mTOR (2983S), NPRL2 (37344S), and pS6K1-Thr389 (9234L) were purchased from Cell Signaling Technology. Antibodies against Ub (sc-8017), LAMP2 (sc-18822), and S6K1 (sc-8418) were purchased from Santa Cruz Biotechnology. Antibodies against KPTN (16094-1-AP), FBXO2 (14590-1-AP), and Flag (20543-1-AP, for immunofluorescence assays) were purchased from Proteintech. Antibodies against RagC (A7479), RagA (A15134), Raptor (A16309), pan–phospho-serine/threonine (AP1475), and β-actin (AC026) were purchased from ABclonal Technology. Antibodies against HA (M20003), Myc (M20002), and Flag (M20008L) were purchased from Abmart. Antibodies against LC3B (ZEN-BIOSCIENCE, 382687) and LAMTOR1 (Solarbio, K006321P) were purchased from the indicated suppliers.

### Plasmids.

The following plasmids were described previously: pCDH-Flag-NPRL2 and pCDH-EGFP-TFEB (29). pBiFC-mCherry-N159 and pBiFC-mCherry-C160 were provided by Xinyue Zhang at Yangzhou University, Yangzhou, China. The coding regions of genes were subcloned into pcDNA3.3-Flag, pcDNA3.3-HA, pCDH-Flag, pCDH-HA, pCDH-Myc, pCDH-EGFP, pGEX-6P-1, pBiFC-mCherry-N159, or pBiFC-mCherry-C160. Oligo DNAs targeting specific genes were synthesized, annealed, and inserted into pLKO.1-puro or pLentiCRISPR-V2. The target sequences of shRNAs and sgRNAs are shown in Supplemental Table 1. The point mutants Ub-K48, Ub-K63, Ub-K48R, Ub-K63R, FBXO2-rsm, FBXO2-Y270/W280A, KPTN-K0, and KPTN-K49/67/262/265R were generated by site-directed mutagenesis. All constructs were verified by sequencing.

### Cell lines.

HEK293T and HeLa cells were obtained from the Cell Bank of Chinese Academy of Sciences (Shanghai, China). HuH-7, SNU449, and SK-Hep1 cells were provided by Zhuan Li (Hunan Normal University) and Ying Zhu (Sun Yat-sen University, Guangzhou, China). All cells were grown in Dulbecco’s modified Eagle medium (DMEM; CellMax) supplemented with 10% fetal bovine serum (FBS; ExCell) and 1% penicillin-streptomycin (Gibco). Cells were maintained at 37°C in a humid incubator with 5% CO_2_.

### Cell treatment and transfection.

For amino acid stimulation, cells were washed with phosphate-buffered saline (PBS), incubated with amino acid–free DMEM (CellMax) for 2 hours (HEK293T, HuH-7, HeLa, and SK-Hep1) or 3 hours (SNU449), and stimulated for the indicated times by directly adding 50× glutamine-free amino acid mixture (Gibco, 11130051) and 50× glutamine (Gibco, 25030081), whose final concentration was the same as that in DMEM. For cancer cell lines, amino acid–free DMEM was supplemented with 40 nM or 100 nM insulin (Sigma). MG132 (Selleck, S2619) was dissolved in DMSO (Sigma, D5879) and used at a concentration of 20 μM for the indicated times.

For cell transfection, cells were transfected with plasmids using the polyethylenimine (PEI; Polysciences) transfection reagent when the cell confluence was about 80%. The transfected cells were harvested 48 hours posttransfection.

### Lentivirus-mediated gene overexpression, knockdown, and knockout.

HEK293T cells were transfected with the lentiviral packaging plasmids (pMD2.G and psPAX2) and lentiviral plasmids expressing cDNA, shRNA, or sgRNA using PEI. At 48 hours posttransfection, supernatant was used to transduce cells in the presence of polybrene (8 μg/mL). At 24 hours posttransduction, the transduced cells were maintained in complete media supplemented with puromycin (1 μg/mL). For generation of knockout cells, puromycin-resistant cells were seeded at a density of 1 cell per well in a 96-well plate and grown for about 2 weeks. The knockout clones were validated by immunoblotting analysis.

### Immunoprecipitation and immunoblotting.

For immunoprecipitation, cells were lysed with NP-40 buffer (0.5% NP-40, 50 mM Tris-HCl [pH 7.4], 150 mM NaCl) or CHAPS buffer (0.3% CHAPS, 40 mM Tris-HCl [pH 7.4], 150 mM NaCl, 10 mM pyrophosphate, 10 mM β-glycerol phosphate, 2.5 mM MgCl_2_) supplemented with EDTA-free protease inhibitor cocktail (Bimake, B14002). Cell lysates were incubated with anti-Flag (Bimake, B26102) or anti-HA (Bimake, B26202) magnetic beads at 4°C for 2–8 hours. The magnetic beads were then washed 3 times with NP-40 buffer containing 400 mM NaCl, and the immunoprecipates were eluted with 1× Laemmli buffer (62.5 mM Tris-HCl [pH 6.8], 0.004% bromophenol blue, 10% glycerol, 5% β-mercaptoethanol, 2% SDS) by boiling at 95°C for 5 minutes.

For immunoblotting, proteins were separated by SDS-PAGE and transferred to nitrocellulose membranes. After blocking with 5% nonfat milk in Tris-buffered saline (TBS) containing 0.05% Tween-20, the membranes were incubated with the indicated antibodies overnight at 4°C. After incubating with horseradish peroxidase–conjugated secondary antibody (Affinity, S0001/S0002), the membranes were visualized with chemiluminescent HRP substrate (FTC, 047-500) using ChemiDoc MP Imaging System (Bio-Rad). The protein band was quantified with the Image Lab software (Bio-Rad).

### Ubiquitination assay.

Cells were lysed with NP-40 buffer containing 0.5% SDS supplemented with EDTA-free protease inhibitors for 20–30 minutes on ice. Then, cell lysates were boiled at 100°C for 10 minutes and diluted 1:2 with NP-40 buffer. Diluted supernatants were immunoprecipitated with anti-Flag or anti-HA magnetic beads. The immunoprecipitates were washed 3 times with NP-40 buffer containing 400 mM NaCl and analyzed by immunoblotting.

### Immunofluorescence assay.

Cells on glass coverslips (Biosharp, BS-14-RC) were fixed with 100% ice-cold methanol (for HeLa cells) or 4% paraformaldehyde solution (for HEK293T cells) for 10 minutes at room temperature, permeabilized with 0.1% Triton X-100 in PBS for 10 minutes, and blocked with 5% BSA in PBS for 1 hour at room temperature. The cells were incubated with the indicated antibodies at 4°C overnight, followed by incubation with Alexa Fluor 488–conjugated (A32723), Alexa Fluor 594–conjugated, (A32740), Texas Red–conjugated secondary antibody against mouse or rabbit (T-2767) (all Invitrogen; 1:1,000) at room temperature for 2 hours, then stained with DAPI (0.5 μg/mL; Solarbio, C0060) for 5–10 minutes at room temperature. Finally, the coverslips were mounted onto slides using an anti-quench mounting buffer (Solarbio, S2100) and then imaged with a fluorescence microscope (Carl Zeiss, Axio Imager M2).

### EGFP-TFEB nuclear translocation assay.

HEK293T or HeLa cells stably expressing EGFP-TFEB were transduced with lentiviruses expressing shCTL or shFBXO2, and KPTN-knockout HEK293T cells stably expressing EGFP-TFEB were transduced with lentiviruses expressing WT KPTN or its mutant KPTN-4KR. At 48 or 72 hours posttransduction, the transduced cells were seeded on glass coverslips, starved with amino acid–free DMEM (supplemented with 100 nM insulin for HeLa cells) for 2 hours, and stimulated with amino acids for 2 hours. The cells were fixed with 4% paraformaldehyde solution for 10 minutes at room temperature, followed by staining with DAPI (0.5 μg/mL) for 5–10 minutes at room temperature. The coverslips were mounted on slides with an anti-quench mounting buffer and imaged with a fluorescence microscope.

### BiFC assay.

HEK293T cells were cotransfected with plasmids pBiFC-mCherry-N159 and pBiFC-mCherry-C160 containing the indicated genes for 36 hours, then seeded on glass coverslips. After 24 hours, cells were fixed with 4% paraformaldehyde for 10 minutes and then stained with DAPI for 5 minutes. Cells were imaged with a fluorescence microscope.

### Recombinant protein expression and purification.

The coding sequence of human KPTN was amplified and inserted into pGEX-6P-1. pGEX-6P-1 or pGEX-6P-1-KPTN was transformed into competent *E*. *coli* BL21(DE3). BL21 bacteria (100 mL culture volume) were grown at 37°C until the culture reached an optical density (OD_600_) of 0.6–0.8, then induced with 0.5 mM isopropyl β-d-thiogalactopyranoside at 16°C for 16 hours. GST fusion proteins were purified using Glutathione Sepharose 4B (GE Healthcare, now Cytiva) according to the manufacturer’s instructions.

### In vitro GST pulldown assay.

KPTN-knockout HEK293T cells transfected with an expression plasmid containing HA-tagged FBXO2 were lysed with NP-40 buffer supplemented with EDTA-free protease inhibitors. WCLs were incubated with 2 μg of purified GST or GST-KPTN protein at 4°C for 4 hours, followed by incubation with glutathione-agarose beads at 4°C for 12 hours. The beads were washed 3 times with NP-40 lysis buffer and eluted with 1× Laemmli buffer by boiling at 95°C for 5 minutes. Samples were subjected to immunoblotting analysis.

### EGFP-LC3B puncta assay.

HeLa cells stably expressing EGFP-LC3B were transduced with lentiviruses expressing the indicated shRNAs. At 72 hours posttransduction, the transduced cells were seeded on glass coverslips. After 16 hours, cells on coverslips were fixed with 100% ice-cold methanol at room temperature for 10 minutes, followed by staining with DAPI (0.5 μg/mL) for 10 minutes. The cells on coverslips were mounted on slides with an anti-quench mounting buffer and imaged with a fluorescence microscope.

### Cell proliferation assay.

HCC cells were seeded at 20,000–50,000 cells per well in 24-well plates. At 12 hours postseeding, HCC cells were transduced with lentiviruses expressing the indicated shRNAs or genes. At the indicated days posttransduction, cells were collected by trypsinization and counted using a hemocytometer (BKMAM).

### Colony formation assay.

Cells were transduced with lentiviruses expressing the indicated shRNAs. At 48 hours posttransduction, the transduced cells were seeded at 1,000 (SNU449) or 700 (HuH-7) cells per well in 6-well plates and grown for 10–15 days with the medium being changed every 2 days. When colonies grew to about 1 mm in diameter, the medium was aspirated, and cells were fixed with 4% paraformaldehyde for 10 minutes. Colonies were stained with 0.1% crystal violet, photographed, and counted.

### Anchorage-independent growth assay.

For the bottom-layer agarose medium, a 1:1 mixture of 1.2% low–melting point agarose (Biosharp, BS144) and DMEM supplemented with 20% FBS was added to 6-well plates (1.5 mL per well) and cooled down at room temperature for solidification. Fifteen thousand (HuH-7) or 20,000 (SK-Hep1) cells were suspended in 1.5 mL warmed-up top-layer agarose (1:1 mixture of 0.8% low–melting point agarose and DMEM supplemented with 40% FBS) for each well. After the top layer was added, the plates were immediately cooled down at 4°C for 10 minutes. Three hundred microliters of complete medium was added onto the top medium every 3 days. At 15–20 days after seeding, colonies were photographed under an inverted microscope and counted.

### Subcutaneous xenograft tumor growth.

Four-week-old male or female BALB/c-Nude mice were obtained from GemPharmatech (Chengdu, China) and housed in a specific pathogen–free animal facility at Hunan Normal University. HuH-7 cells were trypsinized, washed twice with DMEM, and resuspended in DMEM with a density of 5 × 10^6^ cells/mL. Two hundred microliters of the cell suspension was subcutaneously injected into each flank of the mouse. Xenografts were measured with a digital caliper at the indicated days after transplantation, and tumor volume was calculated based on the formula (tumor volume = length × width × width × 0.5). Mice were euthanized when the tumor size reached the upper limit of 1,500 mm^3^. The xenografts dissected from mice were weighed, photographed, and stored at −80°C for further use.

### Protein extraction from mouse xenografts and patient tumors.

Mouse xenografted tumors, liver tumors, and paired adjacent normal tissues from patients were ground with a mortar and pestle in liquid nitrogen and then lysed with RIPA buffer (1 mM EDTA, 1% Triton X-100, 50 mM Tris-HCl [pH 7.4], 150 mM NaCl, 0.1% SDS, 1% sodium deoxycholate) supplemented with EDTA-free protease inhibitor cocktail at 4°C for 4 hours. The lysates were mixed with one-fourth volume of 5× Laemmli buffer and boiled at 95°C for 10 minutes, followed by immunoblotting with indicated antibodies.

### Statistics.

Data were presented as mean ± SEM and analyzed by unpaired 2-tailed Student’s *t* test or 1-way ANOVA followed by Tukey’s multiple comparisons test. All statistical analyses were performed with GraphPad Prism 8. A *P* value of less than 0.05 is considered statistically significant.

### Study approval.

All animal experiments were conducted following the approval of the Animal Use and Care Administration Advisory Committee of Hunan Normal University (2023-671). Liver tumors and paired adjacent normal tissues from patients were all obtained from the third Xiangya Hospital of Central South University. Fresh liver tumor samples were collected from patients and stored at –80°C for further use. This study was approved by the Ethics Committee of the third Xiangya Hospital of Central South University (2023-S052). All patients provided their written informed consent before inclusion in this study.

### Data availability.

The information regarding potential interactions for Supplemental Figure 1A was obtained from the BioGRID database (16). The potential ubiquitination sites of KPTN (Supplemental Figure 4C) were obtained from the PhosphoSitePlus and GPS-Uber databases (22, 23). The mRNA expression data for Supplemental Figure 6A were acquired from the TNMplot database (25). Values for all data points presented in graphs are provided in the Supporting Data Values file. All other data that support the findings of this study are available from the corresponding author.

## Author contributions

JG, JQ, XL, YL, LH, HL, HZ, JZ, PX, and JL performed the experiments. JG, JQ, XL, TL, and SH conceived the study and analyzed the data. JG and SH wrote the manuscript. TL and SH supervised the work and revised the manuscript. JG, JQ, and XL are co–first authors. The authorship order among these authors is assigned according to their contributions.

## Funding support

- National Natural Science Foundation of China (82372673, to SH; 82372247 and 82102387, to TL).
- Natural Science Foundation of Hunan Province (2022JJ20033, to TL).
- Key Project of Education Department of Hunan Province (22A0035, to TL).

## Supplementary Material

Supplemental data

Unedited blot and gel images

Supporting data values

## Version 1 12/16/2025

In-Press Preview

## Version 2 02/16/2026

Electronic publication
