# Supplementary material for: FBXO2-mediated KPTN ubiquitination promotes amino acid–dependent mTORC1 signaling and tumor growth
Source: J Clin Invest. 2025 Dec 16;136(4):e195031. doi: 10.1172/JCI195031 (PMC12904710; doi:10.1172/JCI195031)
Supplement: Supplemental data [file jci-136-195031-s056.pdf]

Supplemental Figures

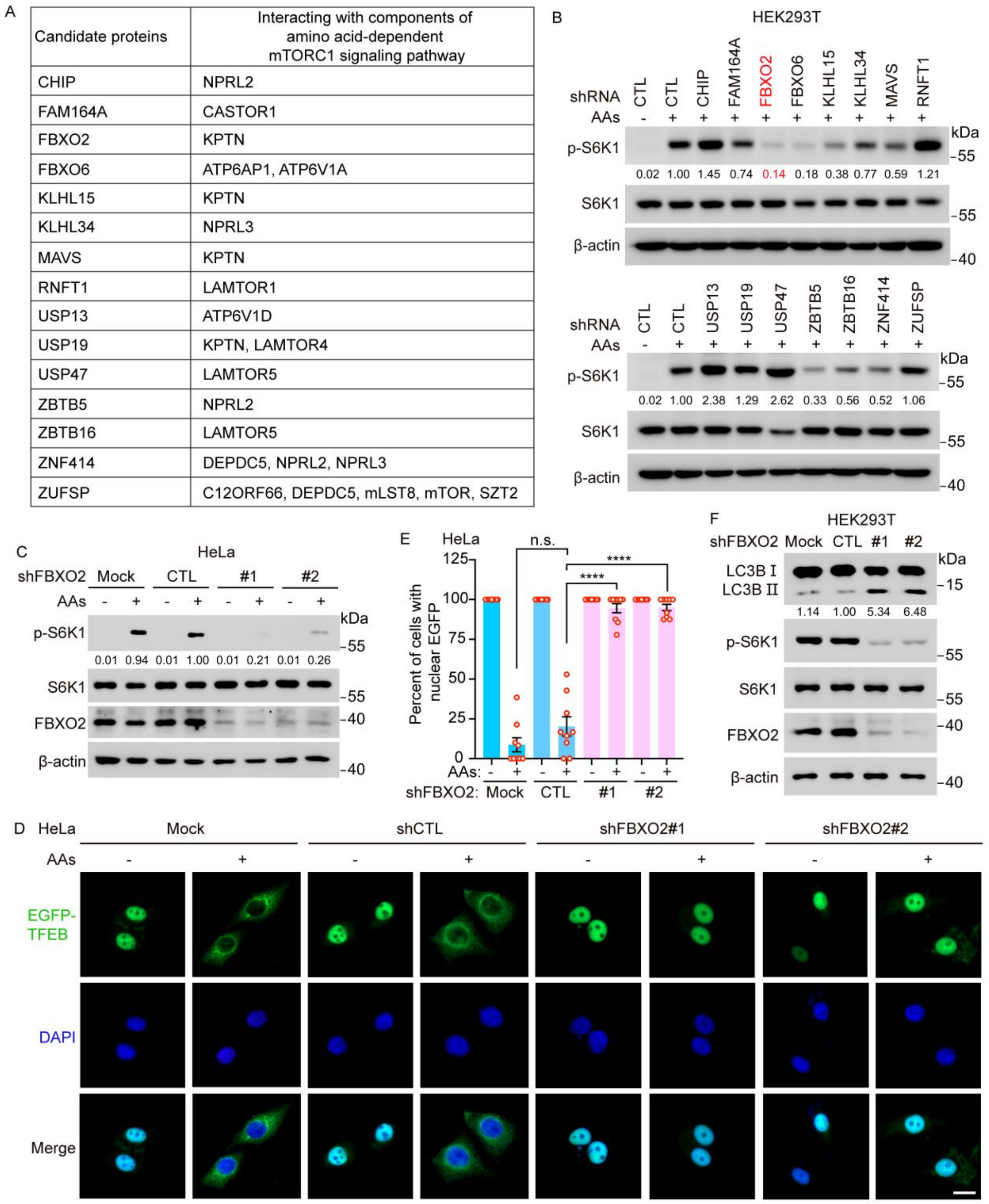

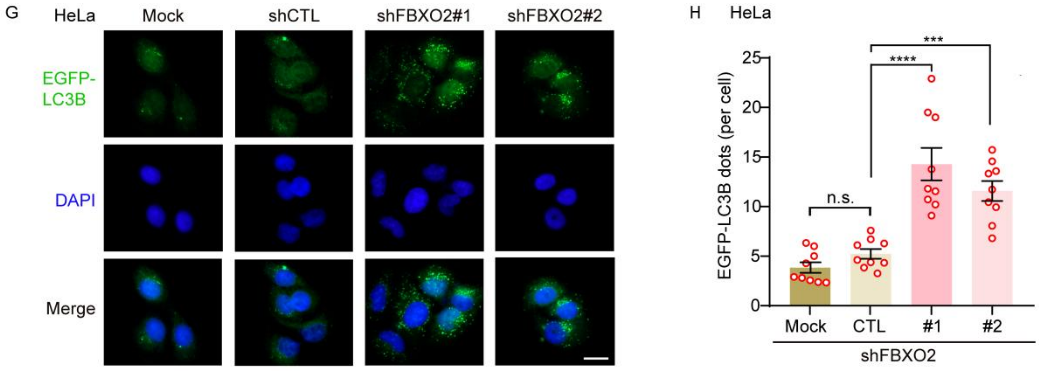

Supplemental Figure 1. FBXO2 promotes amino acid-induced mTORC1 activation. (**A**) List of candidate proteins for screening mTORC1 regulators. (**B** and **C**) HEK293T (B) or HeLa (C) cells stably expressing the indicated shRNAs were deprived of amino acids and serum for 2 h, and then stimulated with amino acids for 20 min. Whole cell lysates (WCLs) were analyzed by immunoblotting with the indicated antibodies. AAs, amino acids. (**D** and **E**) HeLa cells stably expressing EGFP-tagged TFEB (EGFP-TFEB) were transduced with lentiviruses expressing either shCTL or shFBXO2 for 72 h. The transduced cells were deprived of amino acids for 2 h, and then stimulated with amino acids for 2 h, followed by nuclei staining with 4’,6’-diamidino-2-phenylindole (DAPI). Representative images of EGFP-TFEB localization were shown (D) and the quantitative results of EGFP-TFEB localization were present (E). Scale bar, 20 μm. (**F**) WCLs of HEK293T cells transduced with lentiviruses expressing either shCTL or shFBXO2 were analyzed by immunoblotting with the indicated antibodies. (**G** and **H**) HeLa cells stably expressing EGFP-tagged LC3B (EGFP-LC3B) were transduced with lentiviruses expressing either shCTL or shFBXO2, followed by nuclei staining with DAPI. Representative images of LC3 puncta were shown (G) and the quantitative results of LC3 puncta per cell were present (H). Scale bar, 20 μm. Data are presented as means ± SEM, n = 9 independent fields per condition; ns, no significant difference; ***P < 0.001, ****P < 0.0001, one-way ANOVA followed by Tukey’s multiple comparisons test (E and H). Data are representative of at least two independent experiments (C and F).

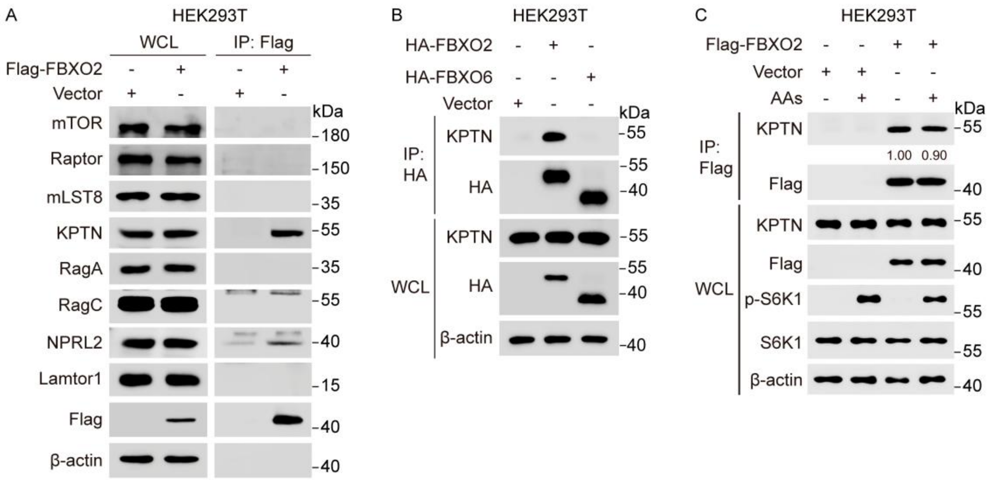

Supplemental Figure 2. FBXO2 interacts with KPTN. (**A** and **B**) WCLs of HEK293T cells stably expressing Flag-tagged FBXO2 (Flag-FBXO2) (A) or HEK293T cells transfected with plasmids expressing the indicated genes (B) were immunoprecipitated with anti-Flag (A) or anti-HA (B) magnetic beads, followed by immunoblotting with the indicated antibodies. (**C**) HEK293T cells stably expressing Flag-FBXO2 were deprived of amino acids and serum for 2 h, and then stimulated with amino acids for 20 min. WCLs were immunoprecipitated with anti-Flag magnetic beads, followed by immunoblotting with the indicated antibodies. Data are representative of at least two independent experiments (A–C).

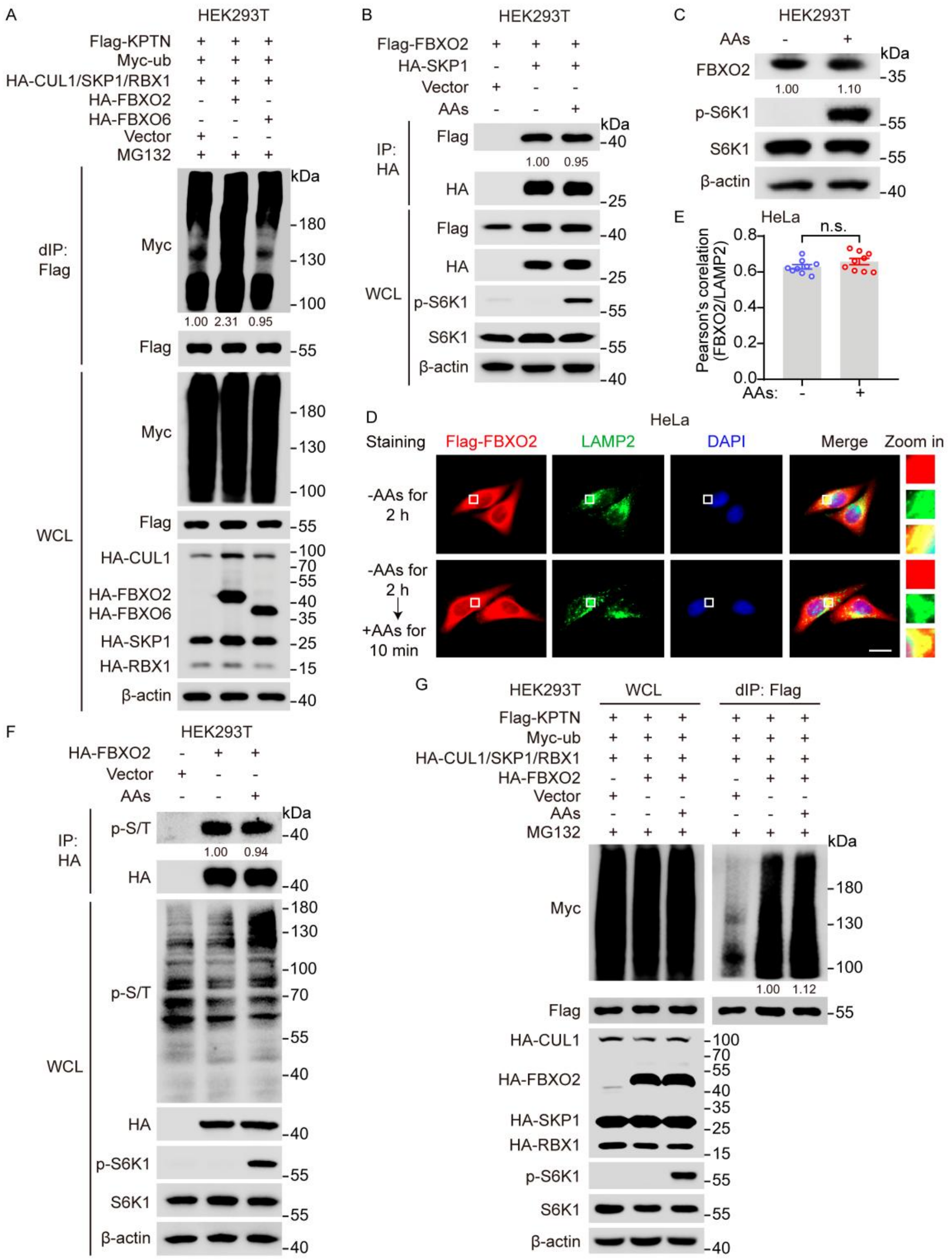

Supplemental Figure 3. FBXO2 constitutively promotes KPTN ubiquitination regardless of amino acid availability. (**A** and **B**) HEK293T cells stably expressing Flag-KPTN (A) or HEK293T cells (B) were transfected with plasmids expressing the indicated genes, and then treated with MG132 (20 μM) for 12 h at 36 h post-transfection (A), or deprived of amino acids and serum for 2 h, and then stimulated with amino acids for 10 min at 46 h post-transfection (B). Denatured WCLs (A) or WCLs (B) were immunoprecipitated with anti-Flag (A) or anti-HA (B) magnetic beads, followed by immunoblotting with the indicated antibodies. (**C**) HEK293T cells were deprived of amino acids and serum for 2 h, and then stimulated with amino acids for 10 min, followed by immunoblotting with the indicated antibodies. (**D** and **E**) HeLa cells stably expressing Flag-FBXO2 were treated as (C),

followed by immunofluorescence analysis with the indicated antibodies. Representative images were shown (D) and co-localization of FBXO2 with LAMP2 was quantified (E). Scale bar, 10 μm. Data are presented as means ± SEM, n = 9 independent fields per condition; ns, no significant difference; unpaired two-tailed Student's t test. (**F** and **G**) HEK293T cells (F) or HEK293T cells stably expressing Flag-KPTN (G) were transfected with plasmids expressing the indicated genes, deprived of amino acids and serum for 2 h, and then stimulated with amino acids for 10 min at 46 h post-transfection. The transfected cells in (G) were also treated with MG132 (20 μM) for 12 h before harvest. WCLs (F) or denatured WCLs (G) were immunoprecipitated with anti-HA (F) or anti-Flag (G) magnetic beads, followed by immunoblotting with the indicated antibodies. Data are representative of at least two independent experiments (A–C, F, and G).

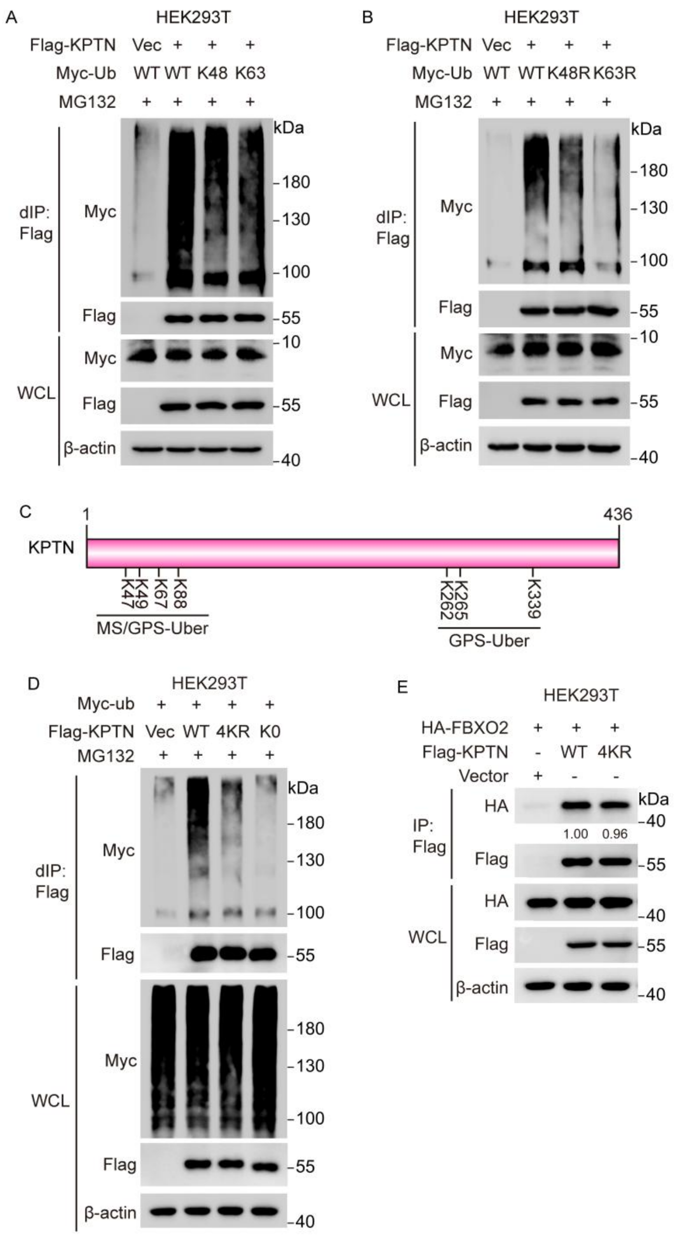

Supplemental Figure 4. KPTN undergoes K48- and K63-linked polyubiquitination at multiple sites. (**A** and **B**) HEK293T cells were transfected with plasmids expressing the indicated genes and treated with MG132 (20 μM) for 4 h at 44 h post-transfection. WT, wild-type; Ub-K48, with the other six lysine residues of Ub mutated to arginine except lysine 48 (K48); Ub-K63, with the other six lysine residues of Ub mutated to arginine except lysine 63 (K63); Ub-K48R, with only lysine 48 of Ub mutated to arginine; Ub-K63R, with only lysine 63 of Ub mutated to arginine. WCLs were denatured and then immunoprecipitated with anti-Flag magnetic beads, followed by immunoblotting with the indicated antibodies. (**C**) Schematic depicting the potential ubiquitination sites of KPTN which are identified by mass spectrum (MS) analysis or predicted by GPS-Uber. (**D**) HEK293T cells stably expressing Myc-Ub were transfected with Flag-tagged wild-type KPTN or its mutants and treated with MG132 (20 μM) for 4 h at 44 h post-transfection. WCLs were

denatured and then immunoprecipitated with anti-Flag magnetic beads, followed by immunoblotting with the indicated antibodies. KPTN-4KR, KPTN-K49/67/262/265R; KPTN-K0, with all the lysine residues of KPTN mutated to arginine. (**E**) WCLs of HEK293T cells transfected with plasmids expressing the indicated genes were immunoprecipitated with anti-Flag magnetic beads, followed by immunoblotting with the indicated antibodies. Data are representative of at least two independent experiments (A, B, D, and E).

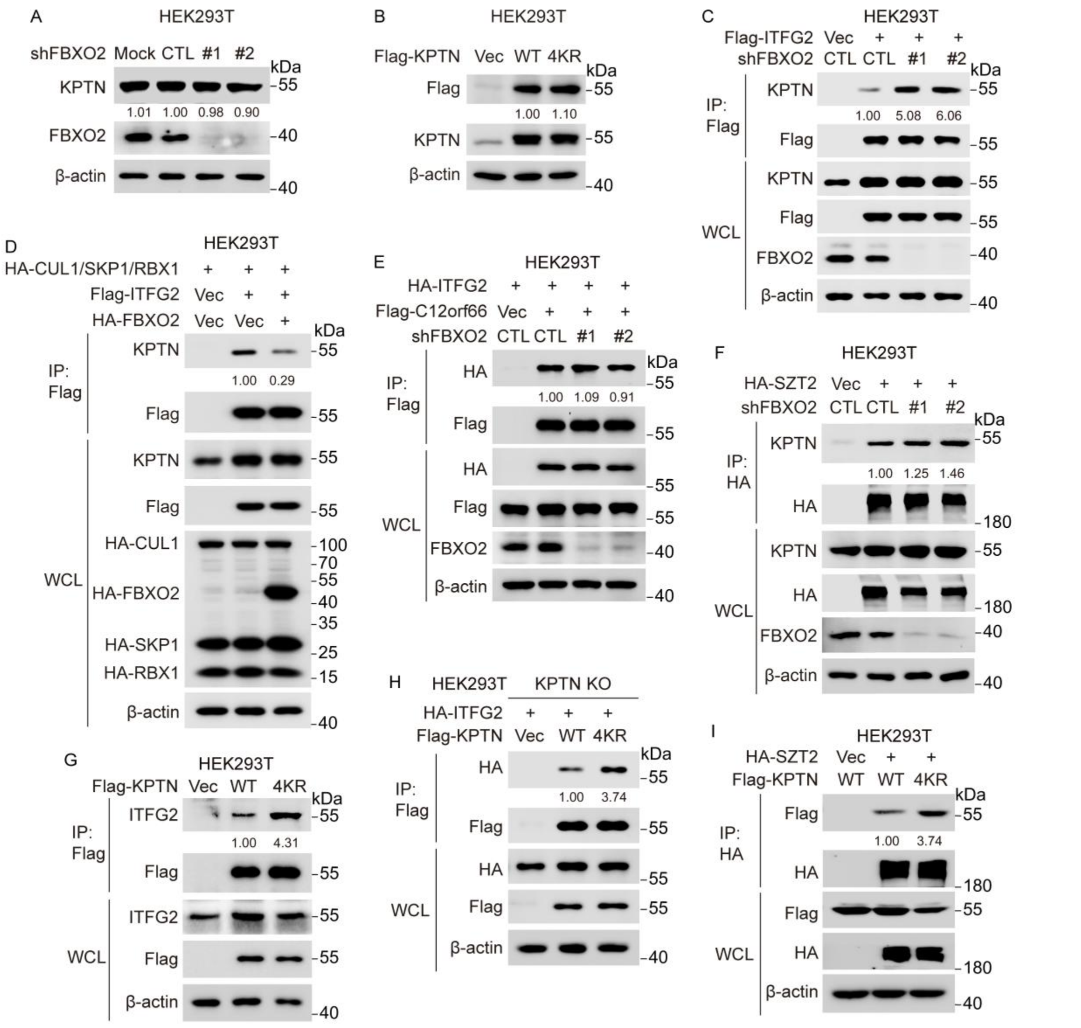

Supplemental Figure 5. FBXO2-mediated KPTN ubiquitination suppresses KICSTOR functions. (**A** and **B**) WCLs of HEK293T cells stably expressing shFBXO2 (A) and Flag-tagged wild-type KPTN or KPTN-4KR mutant (B) were analyzed by immunoblotting with the indicated antibodies. (**C** and **D**) HEK293T cells stably expressing Flag-tagged ITFG2 were transduced with lentiviruses expressing shFBXO2 (C) or transfected with plasmids expressing the indicated genes (D). (**E** and **F**) HEK293T cells stably expressing shFBXO2 were transfected with plasmids expressing the indicated genes. (**G**) HEK293T cells were transduced with lentiviruses expressing Flag-tagged wild-type KPTN or KPTN-4KR. (**H**) KPTN-knockout HEK293T cells stably expressing wild-type KPTN or KPTN-4KR were transduced with lentiviruses expressing HA-tagged ITFG2. (**I**) HEK293T cells were transfected with plasmids expressing the indicated genes. WCLs (C–I) were immunoprecipitated with anti-Flag (C–E, G, and H) or anti-HA (F and I) magnetic beads, followed by immunoblotting with the indicated antibodies. Data are representative of at least two independent experiments (A–I).

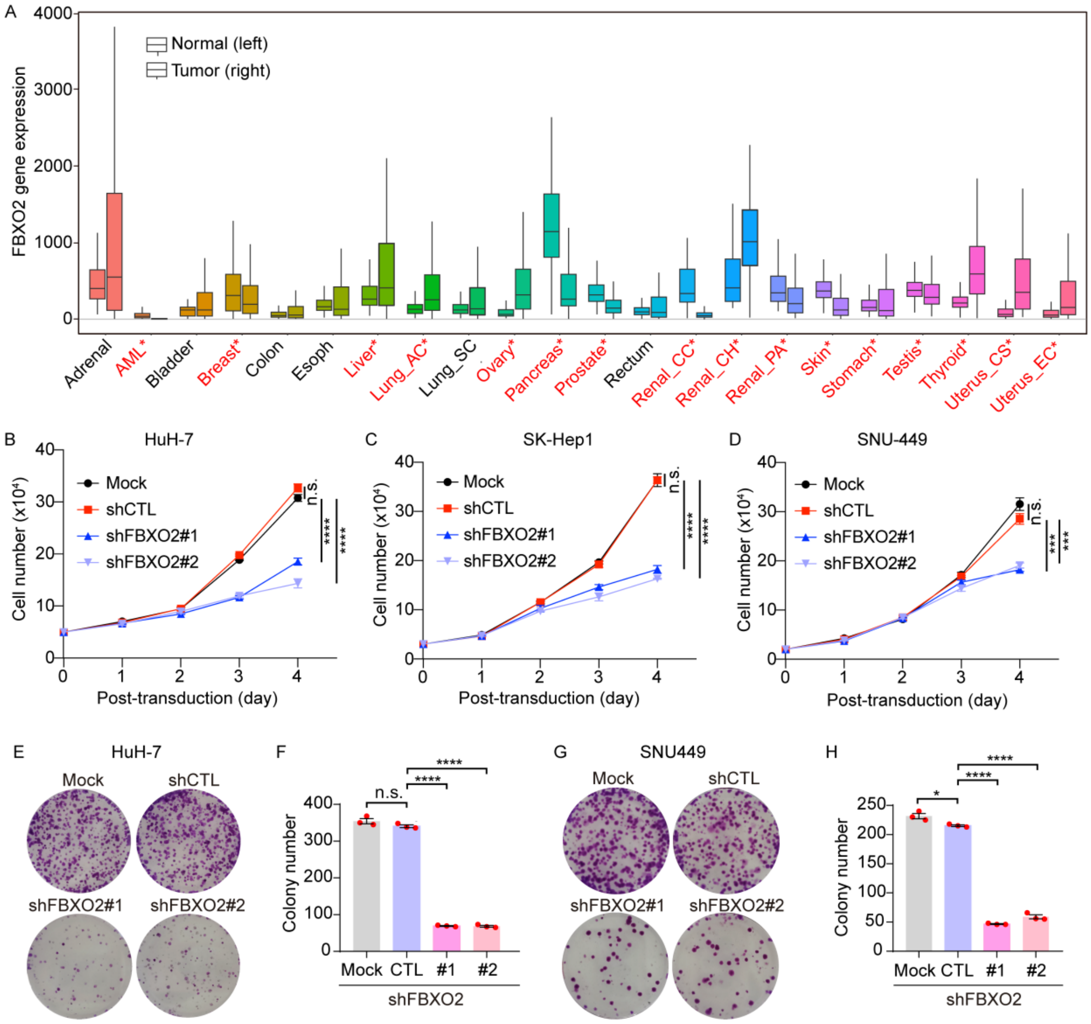

Supplemental Figure 6. FBXO2 promotes the proliferation of hepatocellular carcinoma cells. (**A**) The expression of FBXO2 in 22 types of normal and cancer tissues was analyzed using TNMplot database. Red*, Mann-Whitney p<0.05 and expression >10 in tumor or normal samples. (**B**–**D**) HuH-7 (B), SK-Hep1 (C), and SNU449 (D) cells were transduced with lentiviruses expressing shCTL or shFBXO2 for 24 h and then seeded in 24-well pates. Cell numbers were counted for 4 consecutive days. (**E**–**H**) HuH-7 (E and F) and SNU449 (G and H) cells were transduced with lentiviruses expressing shCTL or shFBXO2 for 48 h and then seeded in 6-well pates. Colonies were photographed (E and G) and counted (F and H) at 15 days post-seeding. Data are presented as means ± SEM, n = 3 biologically independent repeats; ns, no significant difference; *P < 0.05, ***P < 0.001, ****P < 0.0001, one-way ANOVA followed by Tukey’s multiple comparisons test (B–D, F, and H).

Supplemental Tables

Supplemental Table 1. List of target sequences of sgRNAs and shRNAs and primers for mutagenesis

**sgRNA target sequences**	
sgGFP	5’-GCTGAAGCACTGCACGCCGT-3’
sgFBXO2	5’-GGTGGGAGACACCAGCGGGA-3’
sgKPTN	5’-ATAAGCTTGTTCTGCAGCTC-3’
sgNPRL2	5’-ATAAGCTTGTTCTGCAGCTC-3’
**shRNA target sequences**	
shCTL	5’-CCTAAGGTTAAGTCGCCCTCG-3’
shCHIP#1	5’-GAAGAGGAAGAAGCGAGACAT-3’
shCHIP#2	5’-CCCAAGTTCTGCTGTTGGACT-3’
shFAM164A#1	5’-GCCAAGTTCAGAGTTTATGAT-3’
shFAM164A#2	5’-CTTCCCTTAATGGTGGAAATA-3’
shFBXO2#1	5’-TGGTGTGACGTGGAGCATGGT-3’
shFBXO2#2	5’-TCGTGGTGAAGGACTGGTACT-3’
shFBXO6#1	5’-GAGGAGCTACTAGACACATTC-3’
shFBXO6#2	5’-TGTGCTGAAGAGGATATGTTT-3’
shKLHL15#1	5’-GCGTAAACATCGAGGGAG-3’
shKLHL15#2	5’-CTGAGTATGAATACCGTTCAT-3’
shKLHL34#1	5’-GGTTGGAGAGGCGCTAGATTT-3’
shKLHL34#2	5’-TGGCTATAAAGGCTGTAATTA-3’
shMAVS#1	5’-CCAGAGGAGAATGAGTATAAG-3’
shMAVS#2	5’-TTTACCAAGGGTTGGATATAT-3’
shRNFT#1	5’-CTGATGATACTGCCGCAGAAT-3’
shRNFT#2	5’-GCCTTCTTTCATCATGCCTTT-3’
shUSP13#1	5’-CTGTGTACTTCACTGGAAA-3’
shUSP13#2	5’-CGATTTAAATAGCGACGATTA-3’

shUSP19#1	5’-CTCCACTGCGAGCGAAGTATT-3’
shUSP19#2	5’-GATCAATGACTTGGTGGAGTT-3’
shUSP47#1	5’-GCAGCTTTCAAACAACATTTA-3’
shUSP47#2	5’-GTCACTTCTCGACGCTAATTT-3’
shZBTB5#1	5’-CCAGGAAGATAGTGCGATCAT-3’
shZBTB5#2	5’-CTCTGTTGTTAAGGCATGTAA-3’
shZBTB16#1	5’-ATCAGCTGGAGACGCACTATA-3’
shZBTB16#2	5’-AGATCCTCTTCCACCGCAATA-3’
shZNF414#1	5’-ACCGTTCCTGCCCTACTTGAA-3’
shZNF414#2	5’-ACAGCAAACTGCACTACAAAC-3’
shZUFSP#1	5’-GAAGCACTTCATAGGTATTAT-3’
shZUFSP#2	5’-GCAAGAAGAAGACAGAAAGAG-3’
