# Supplementary material for: FBXO2-mediated KPTN ubiquitination promotes amino acid–dependent mTORC1 signaling and tumor growth
Source: J Clin Invest. 2025 Dec 16;136(4):e195031. doi: 10.1172/JCI195031 (PMC12904710; doi:10.1172/JCI195031)

Full unedited blot for Figure 1A

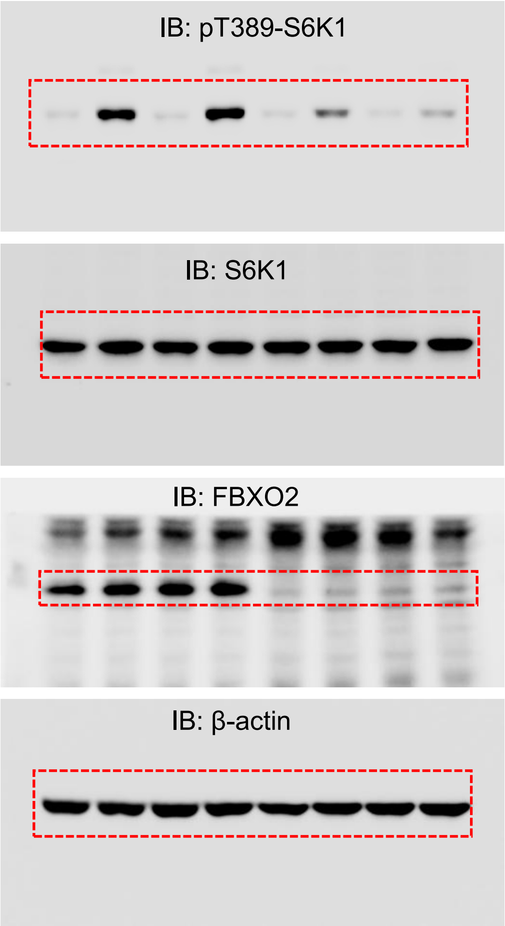

Full unedited blot for Figure 1B

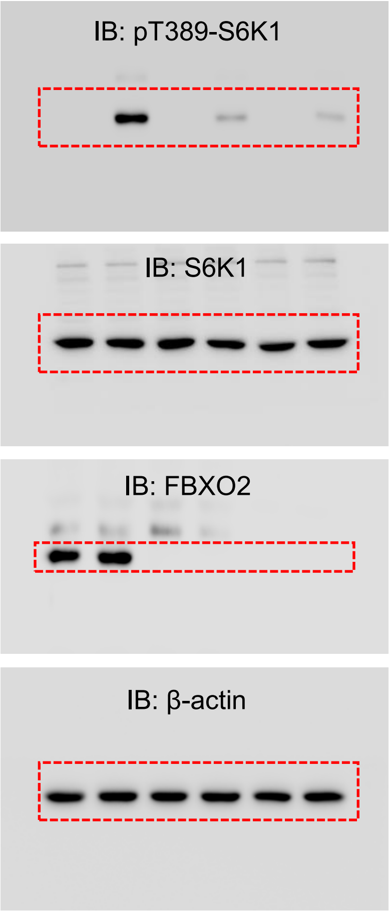

Full unedited blot for Figure 1C

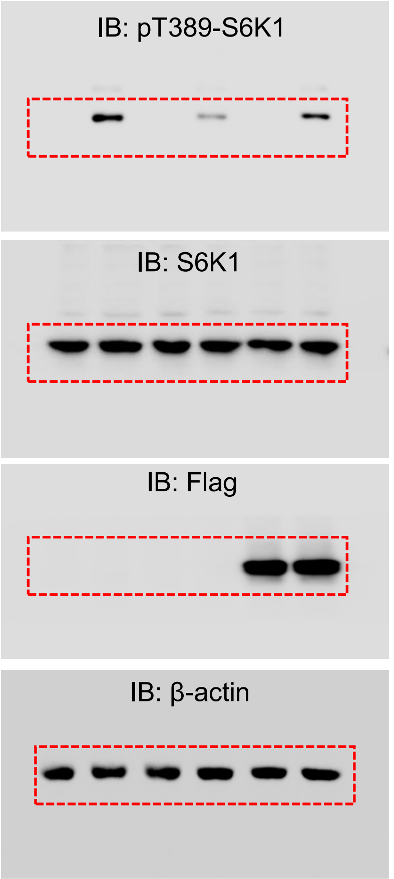

Full unedited blot for Figure 2A

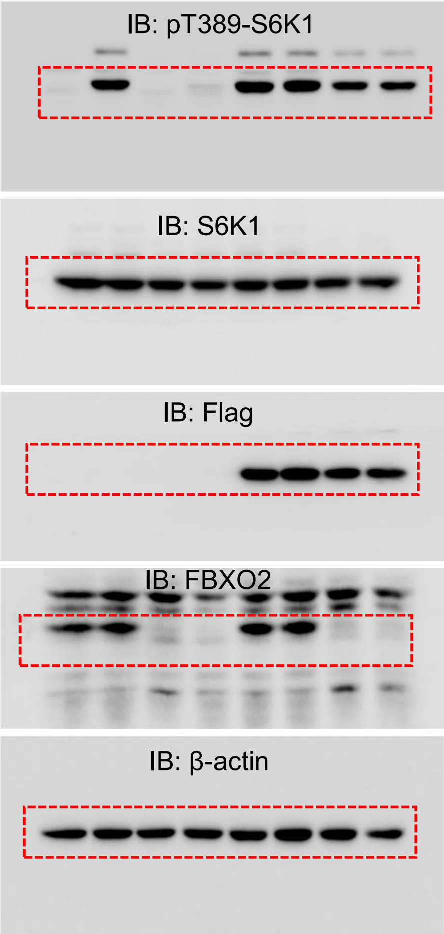

Full unedited blot for Figure 2B

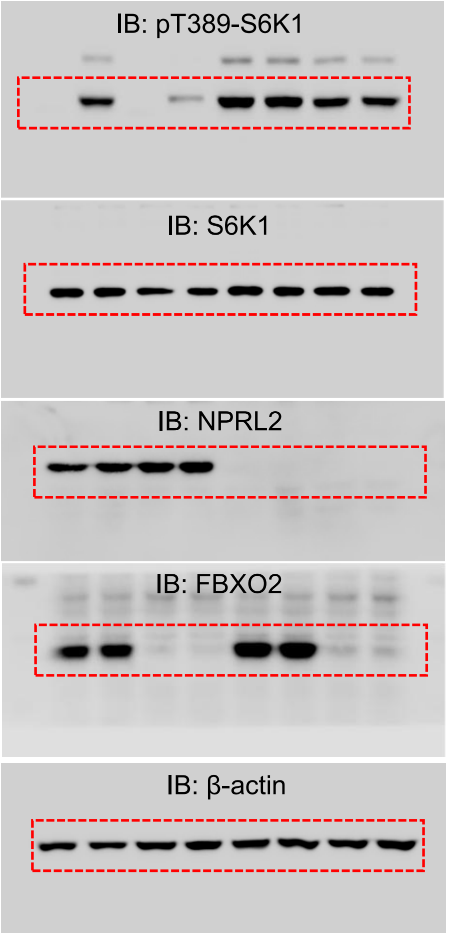

Full unedited blot for Figure 2C

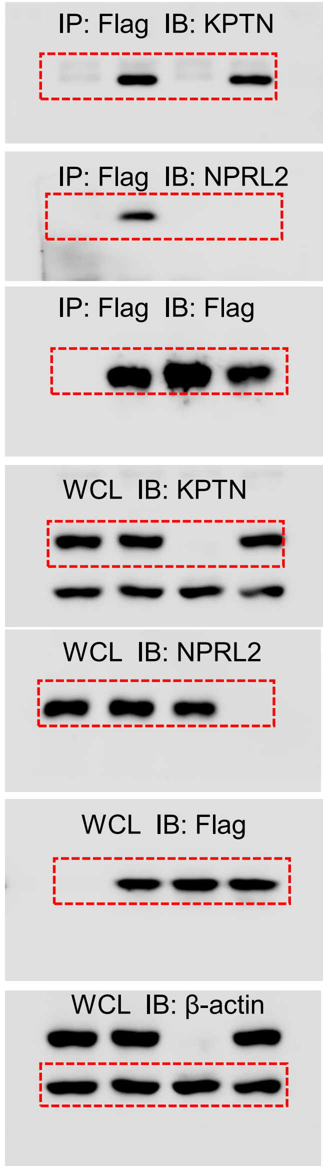

Full unedited blot for Figure 2D

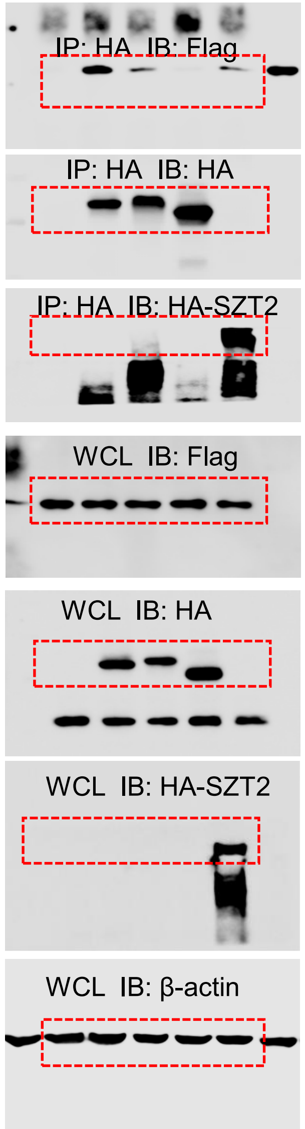

Full unedited blot for Figure 2E

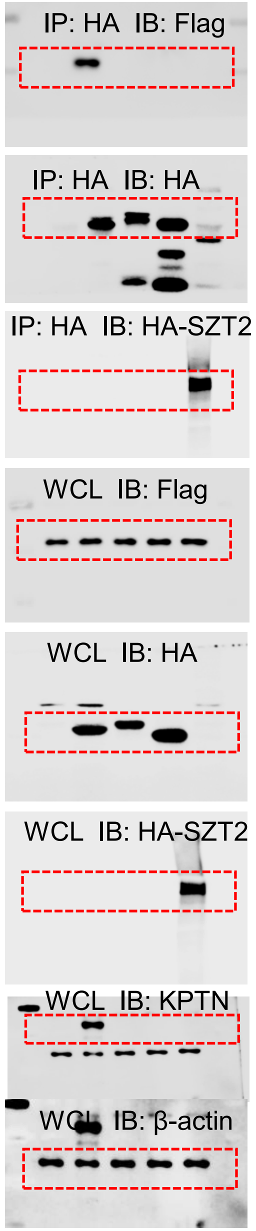

Full unedited blot for Figure 2K

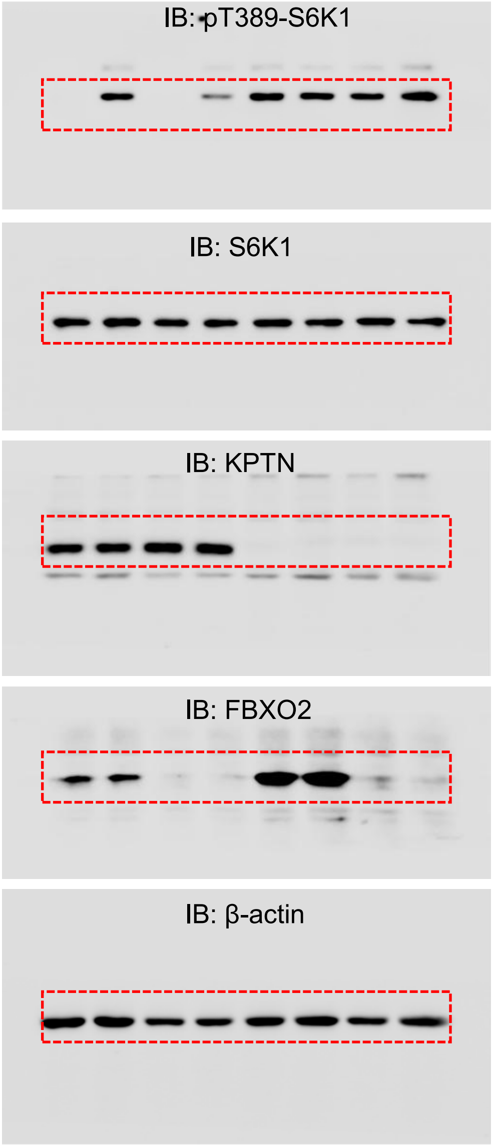

Full unedited blot/gel for Figure 2I

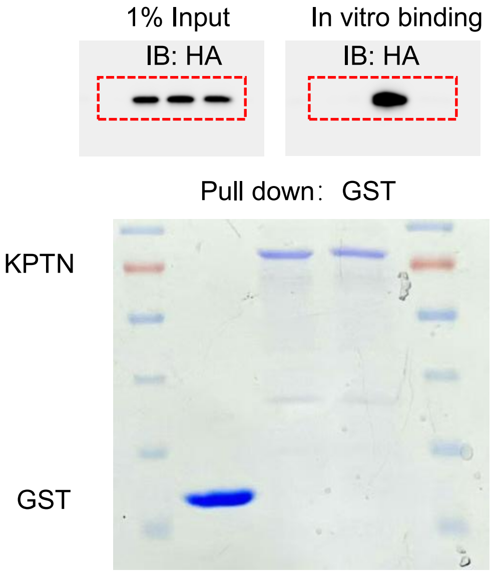

Full unedited blot for Figure 3A

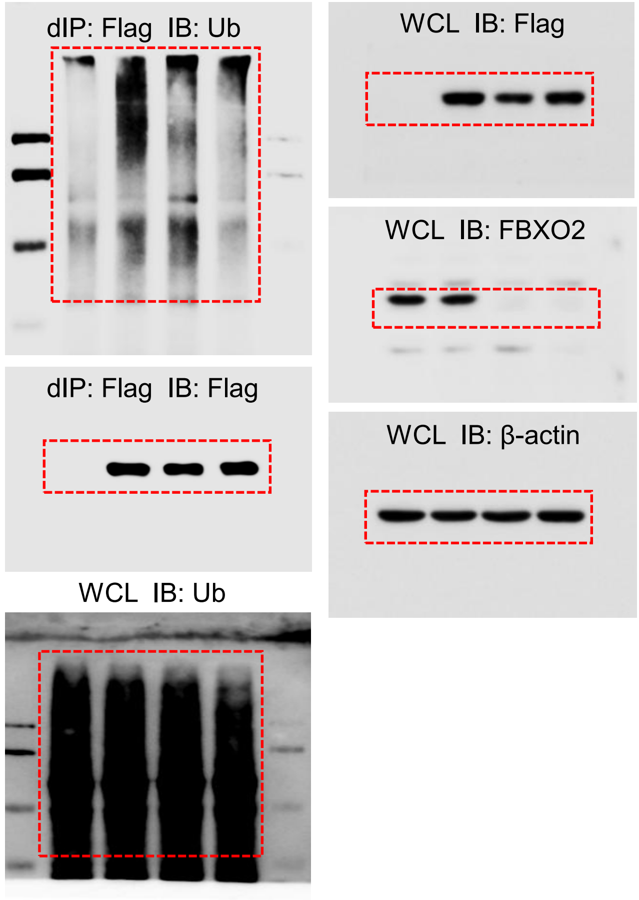

Full unedited blot for Figure 3B

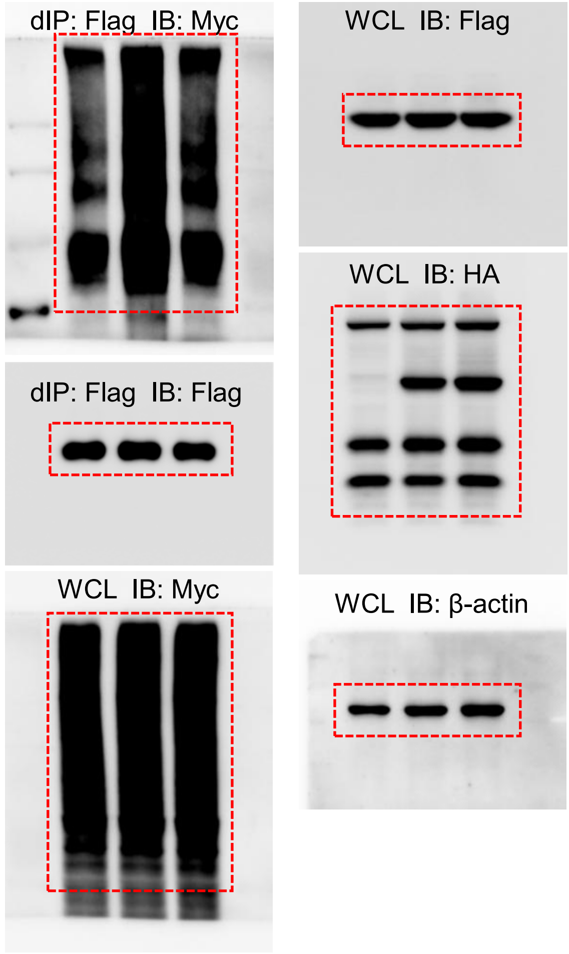

Full unedited blot for Figure 3C

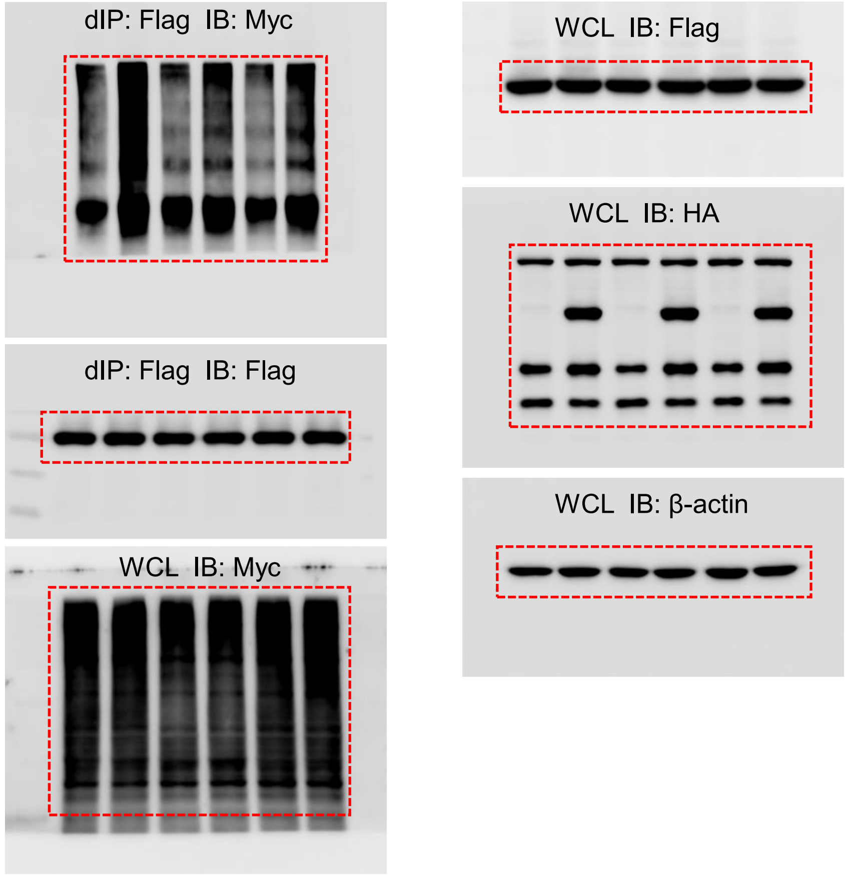

Full unedited blot for Figure 3D

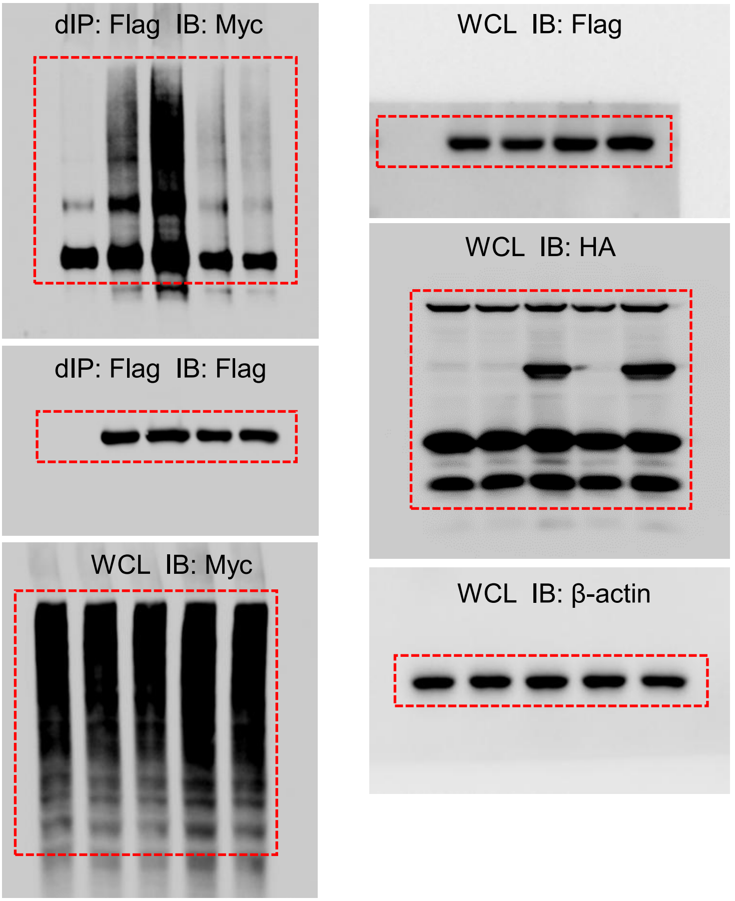

Full unedited blot for Figure 3G

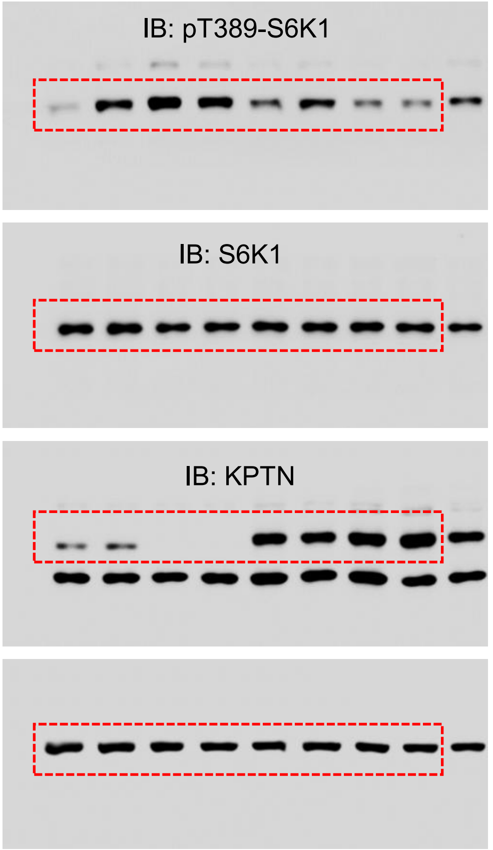

Full unedited blot for Figure 4A

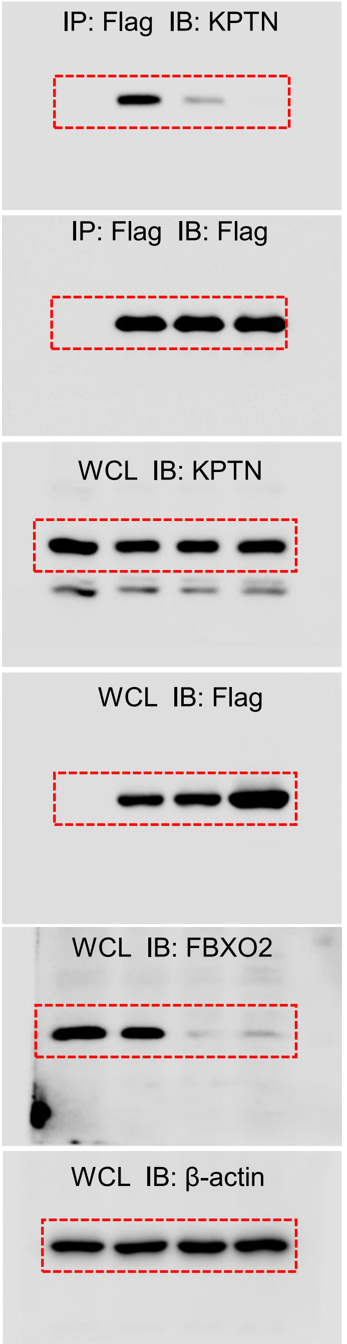

Full unedited blot for Figure 4B

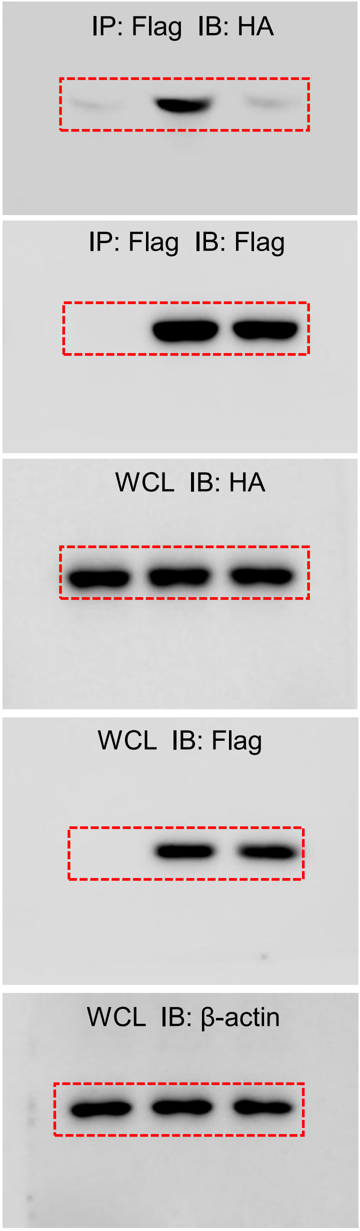

Full unedited blot for Figure 4C

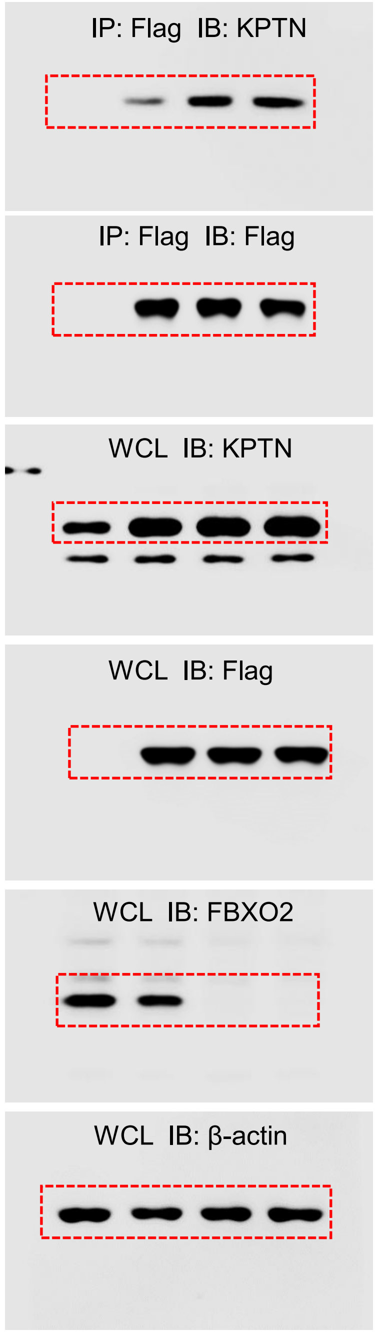

Full unedited blot for Figure 4D

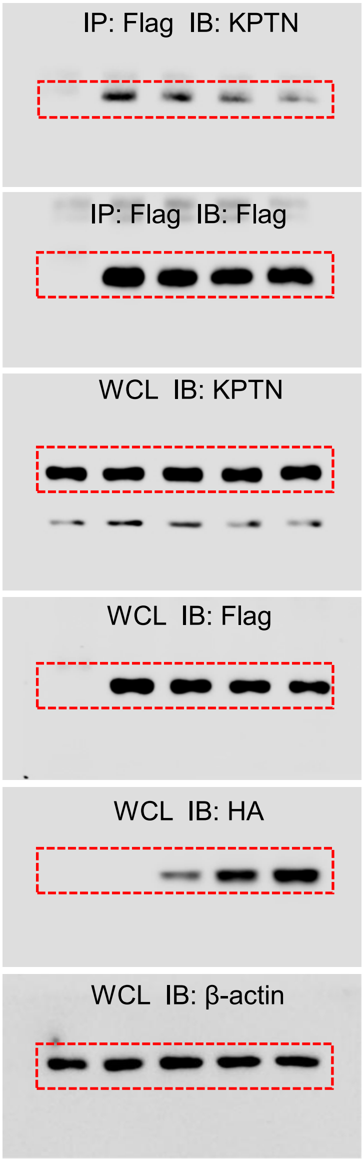

Full unedited blot for Figure 4G

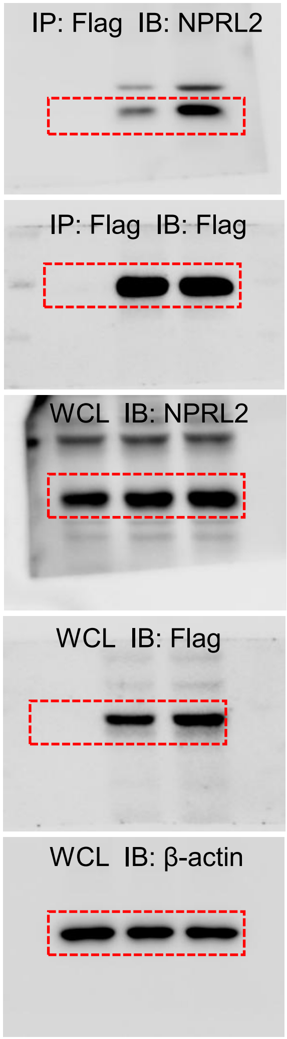

Full unedited blot for
Figure 4J

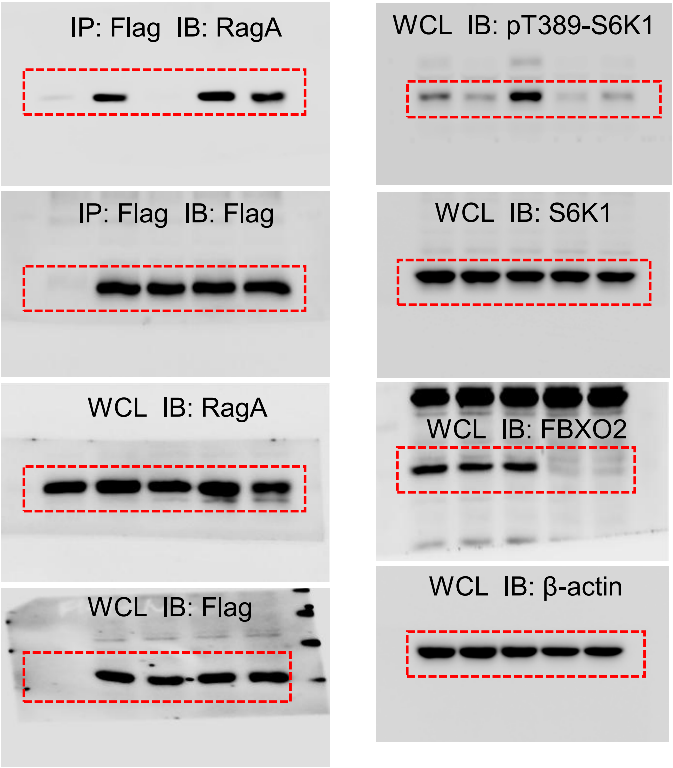

Full unedited blot for
Figure 4K

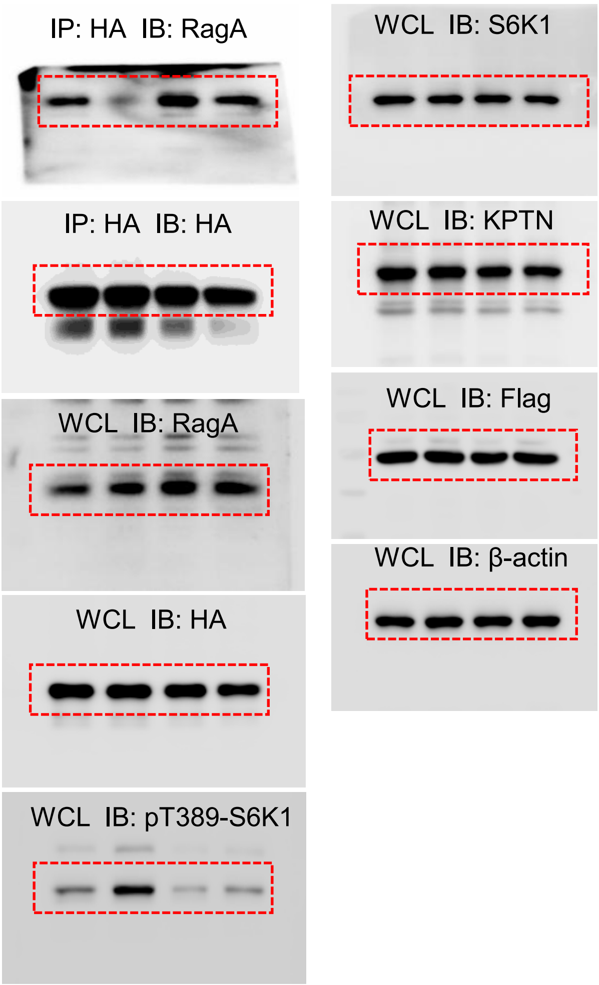

Full unedited blot for Figure 5A

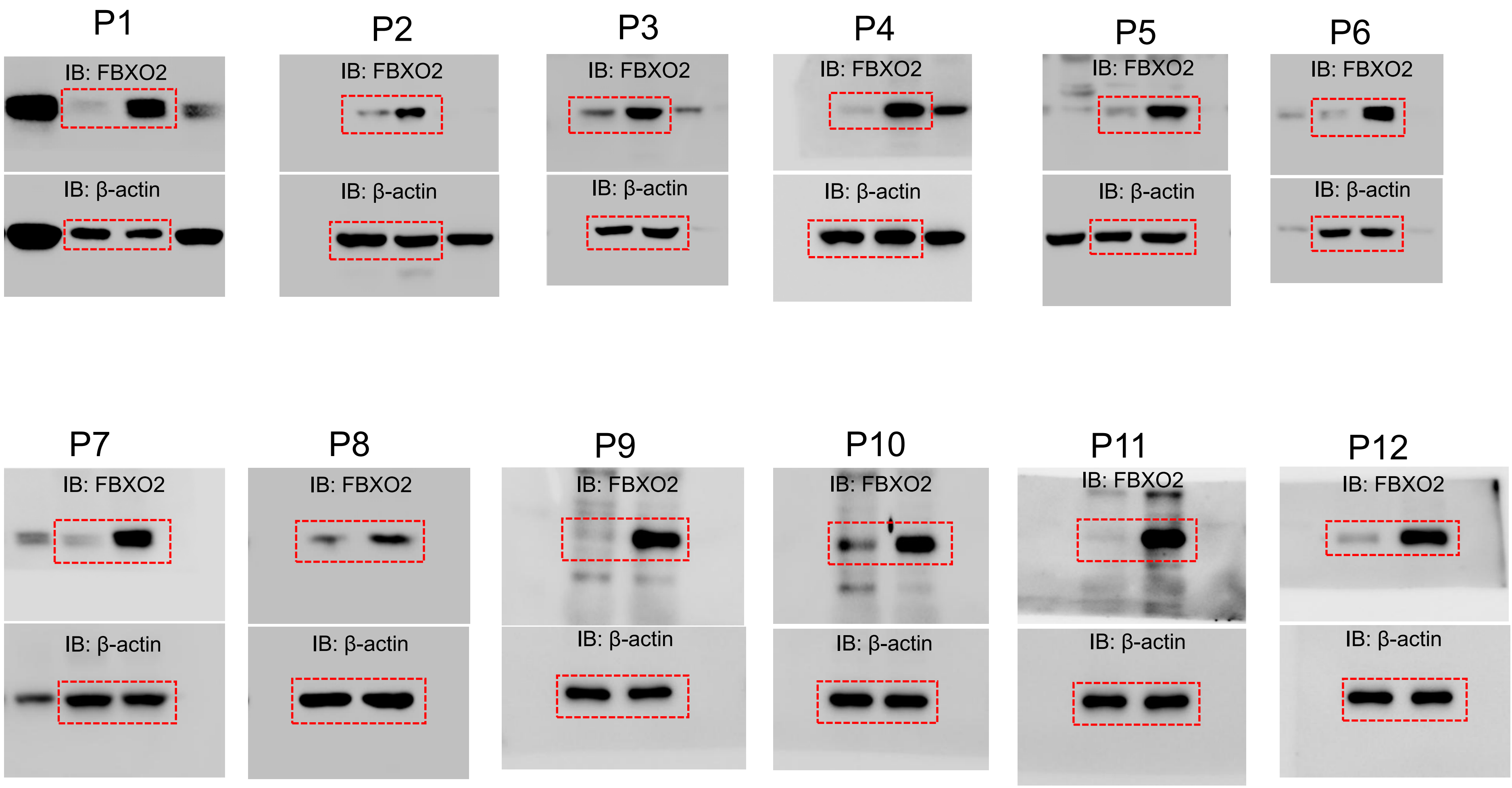

Full unedited blot for Figure 5C

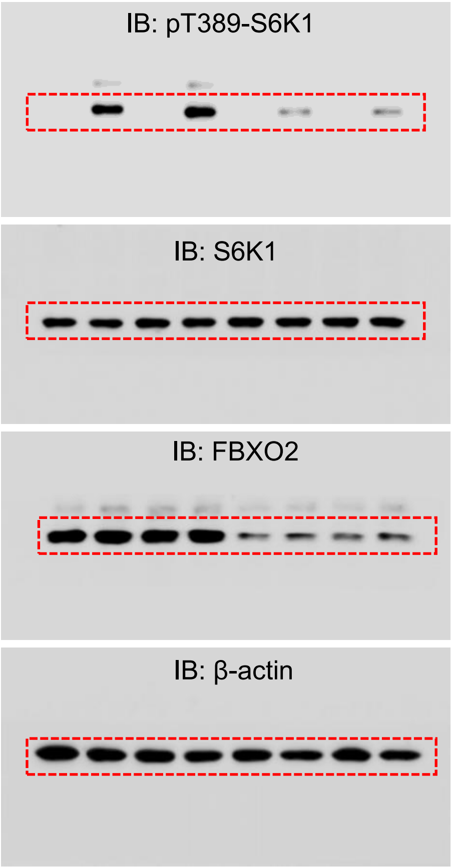

Full unedited blot for Figure 5D

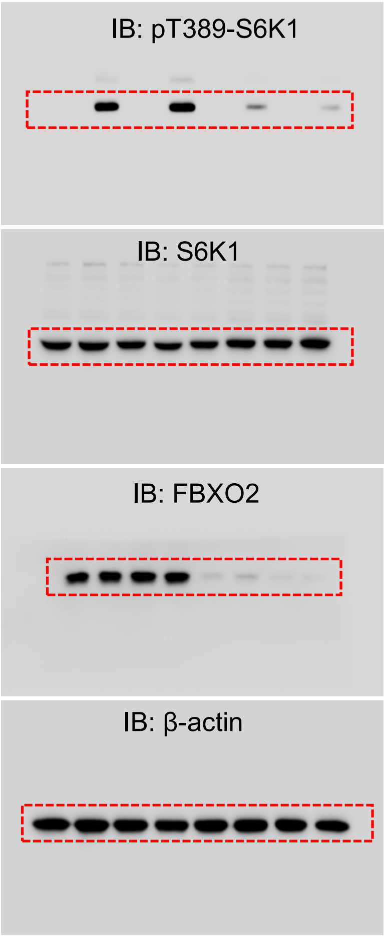

Full unedited blot for Figure 5E

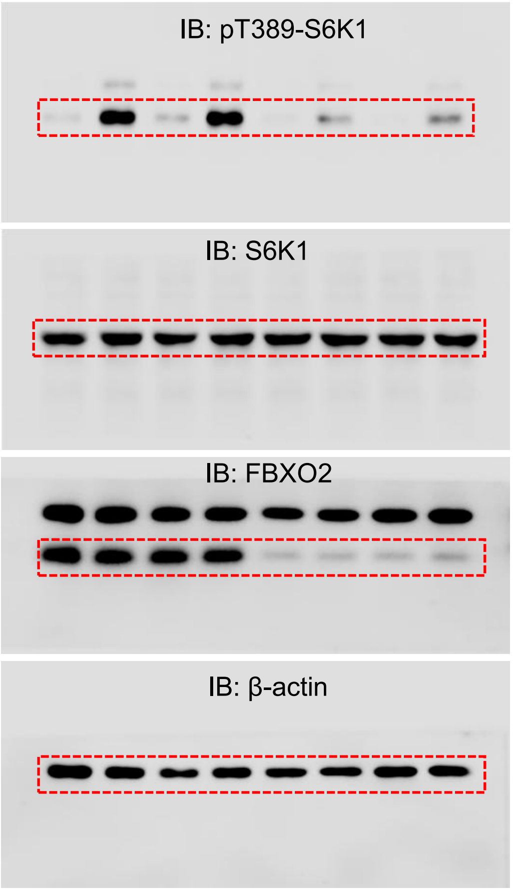

Full unedited blot for Figure 5M

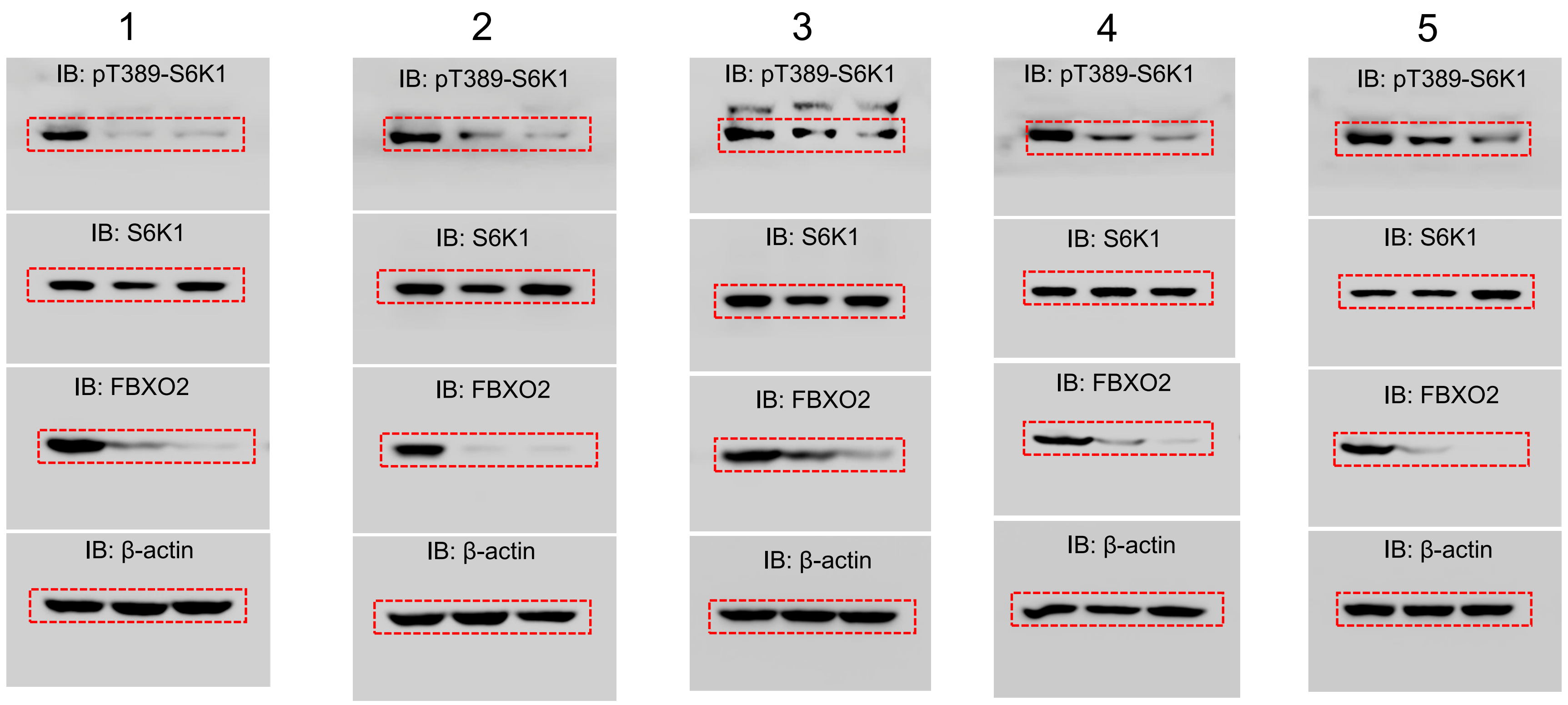

Full unedited blot for Figure 5M

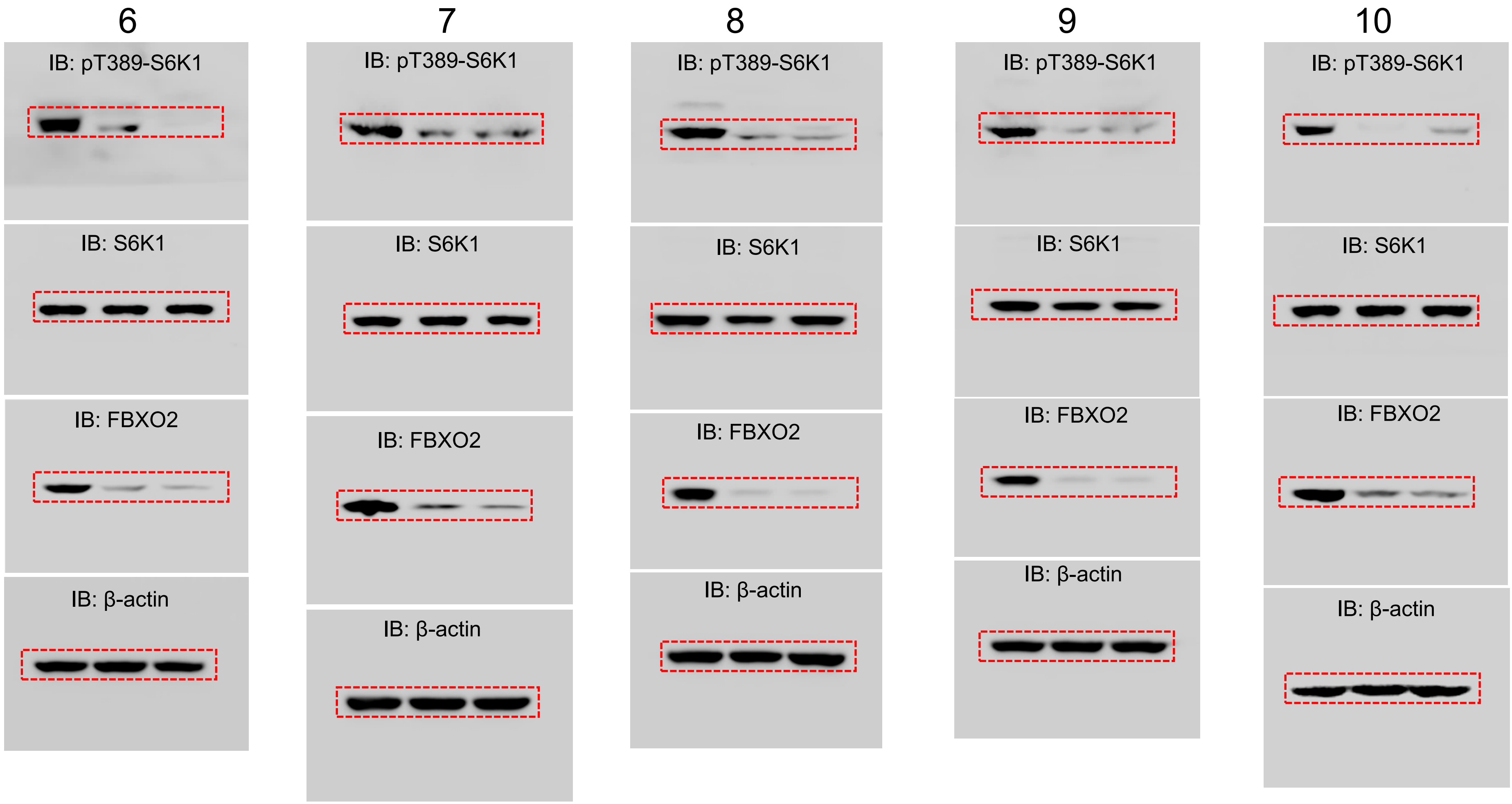

Full unedited blot for Figure 6A

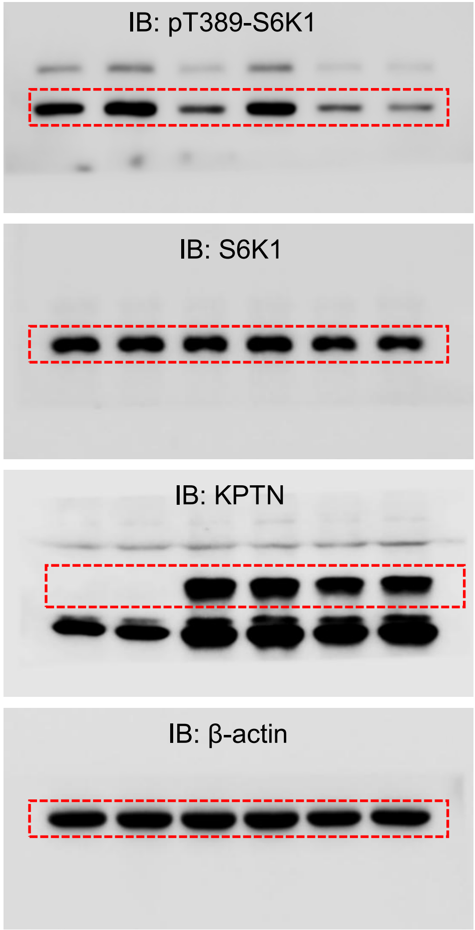

Full unedited blot for
Figure 6H

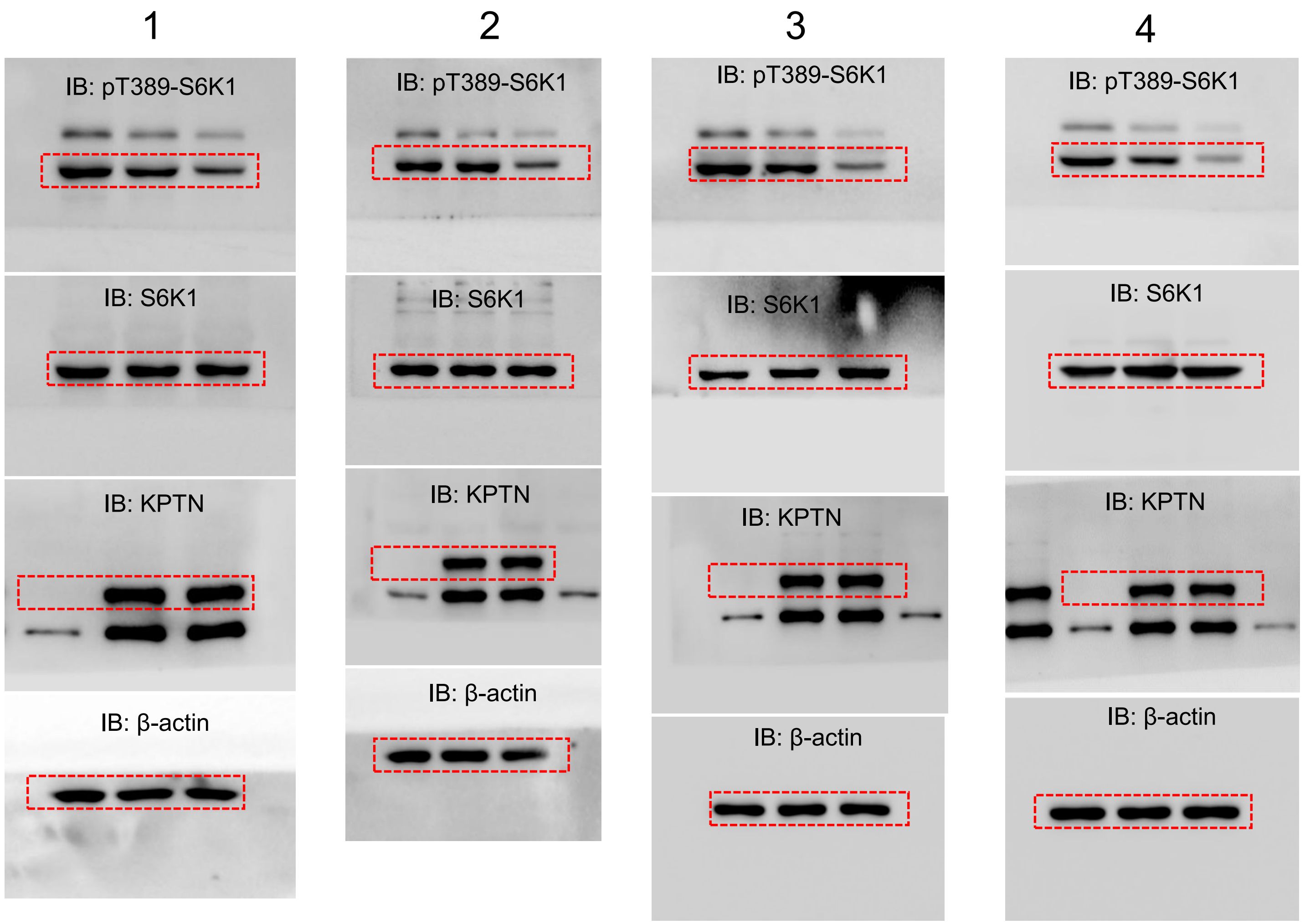

Full unedited blot for
Figure 6H

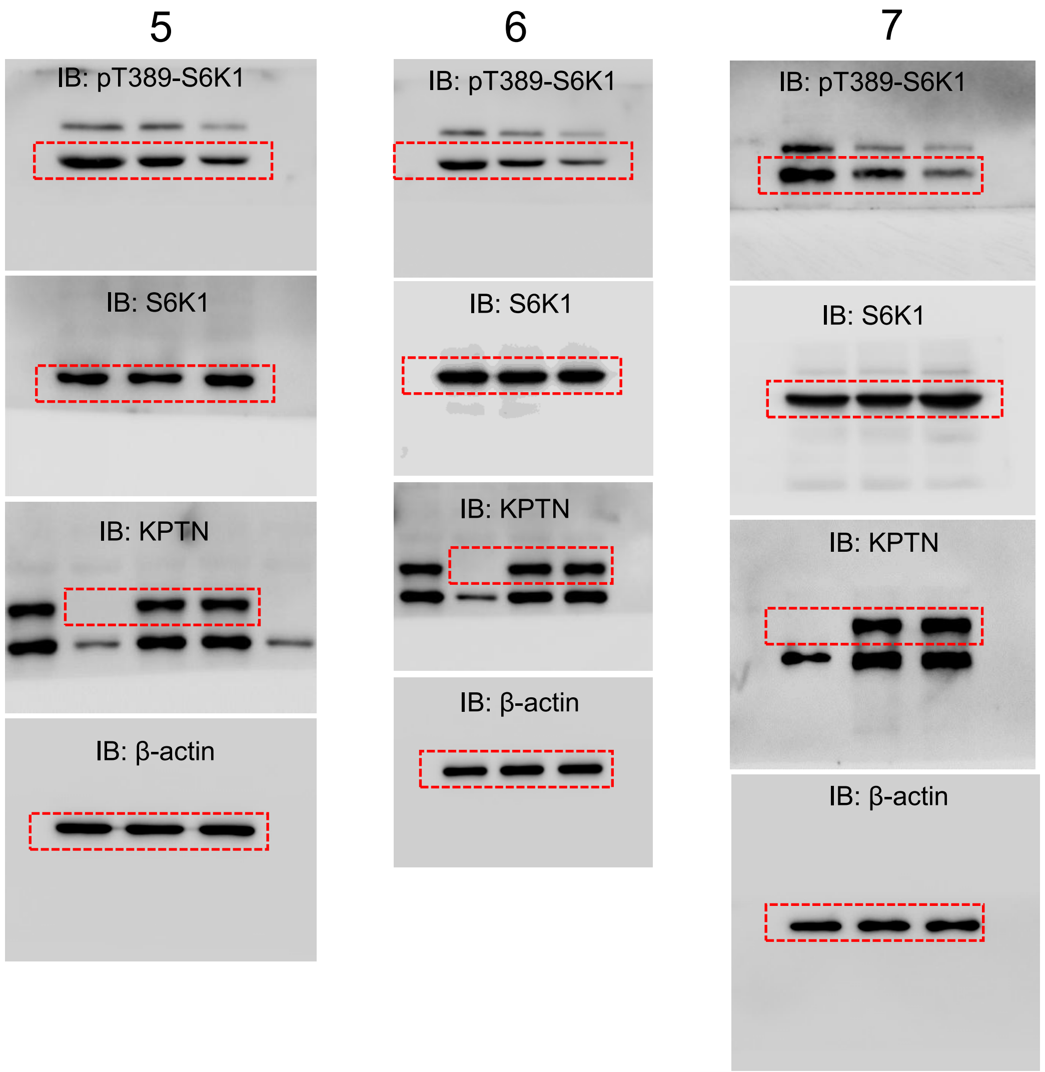

Full unedited blot for
Supplemental Figure 1B

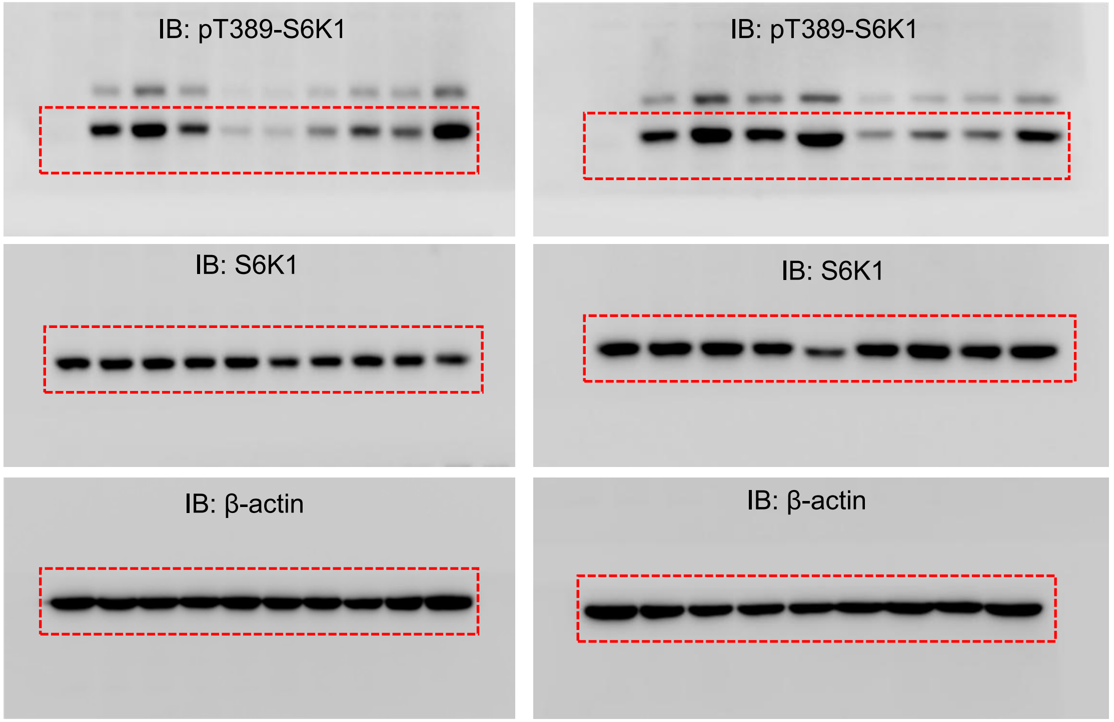

Full unedited blot for
Supplemental Figure 1C

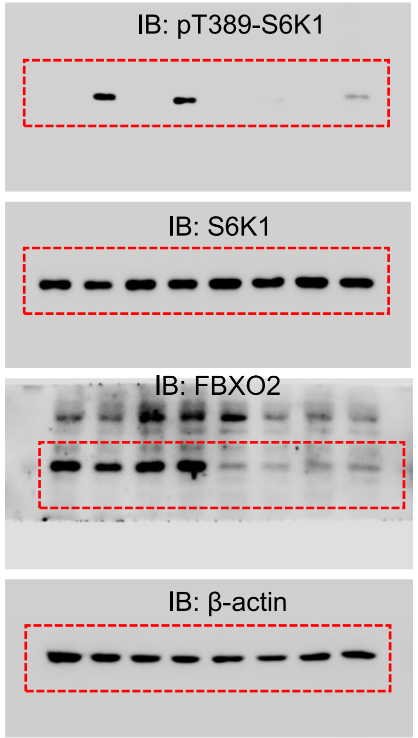

Full unedited blot for
Supplemental Figure 1F

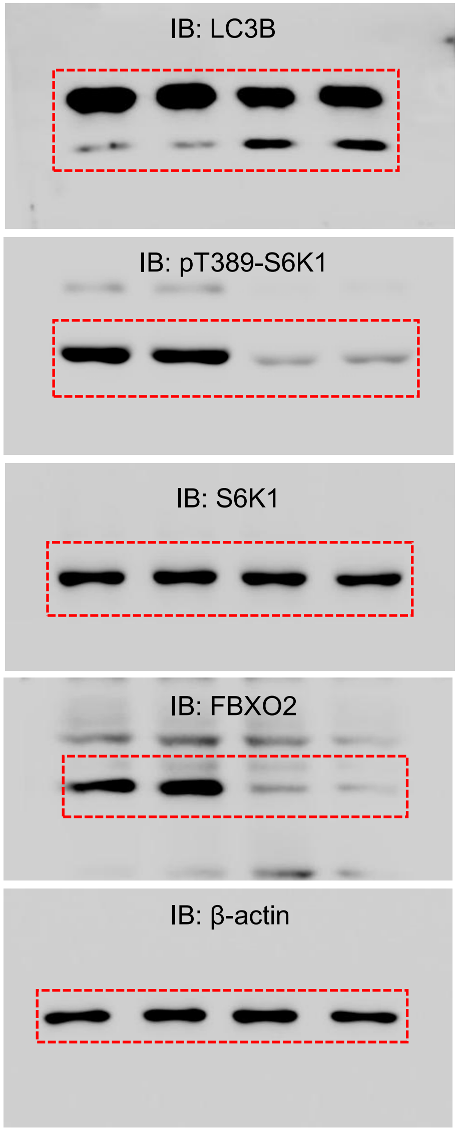

Full unedited blot for Supplemental Figure 2A

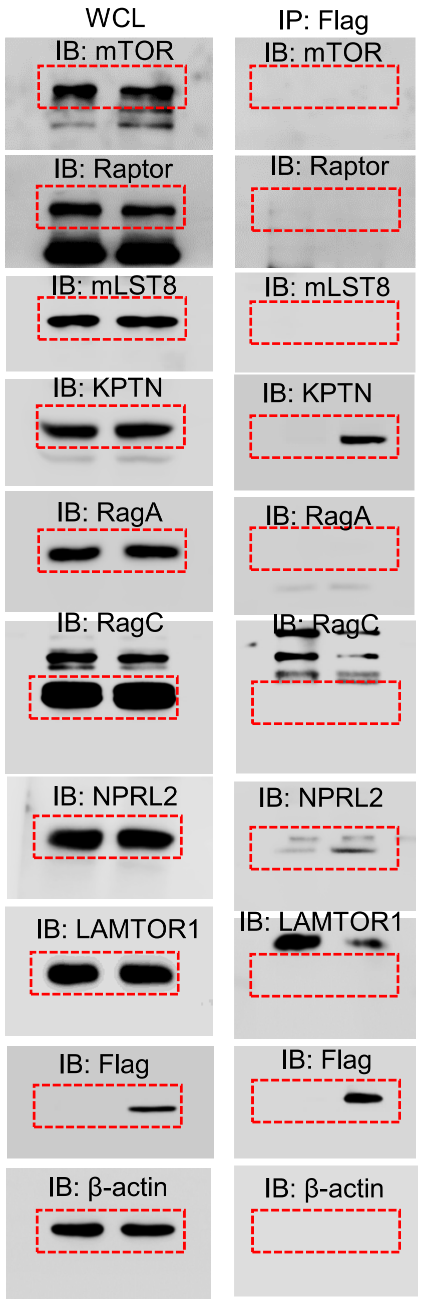

Full unedited blot for Supplemental Figure 2B

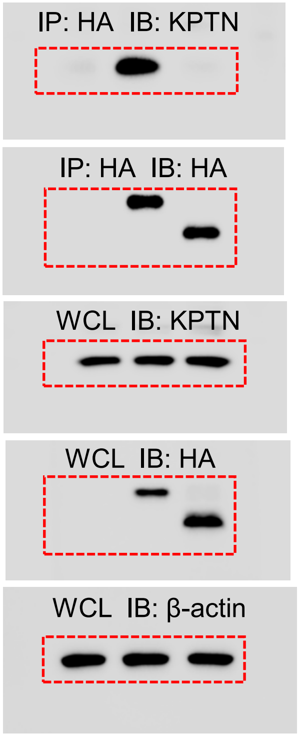

Full unedited blot for Supplemental Figure 2C

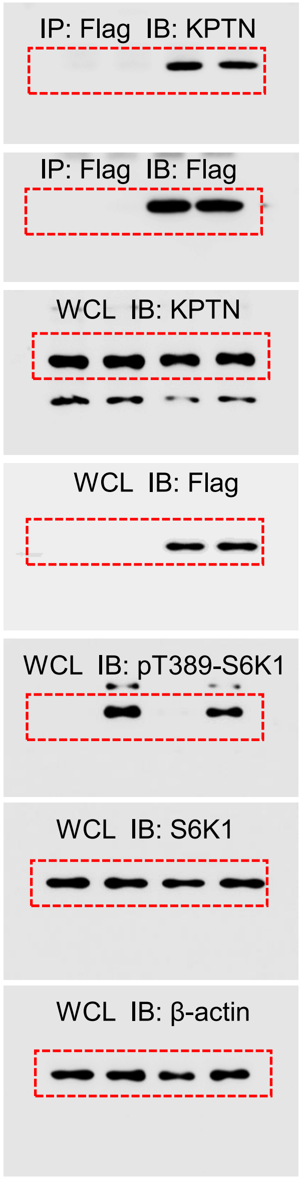

Full unedited blot for
Supplemental Figure 3A

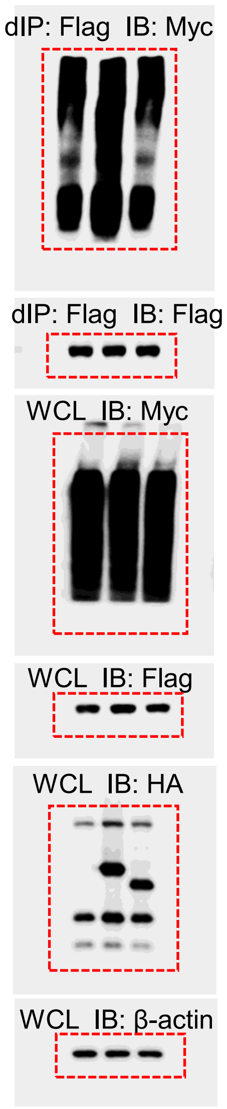

Full unedited blot for
Supplemental Figure 3B

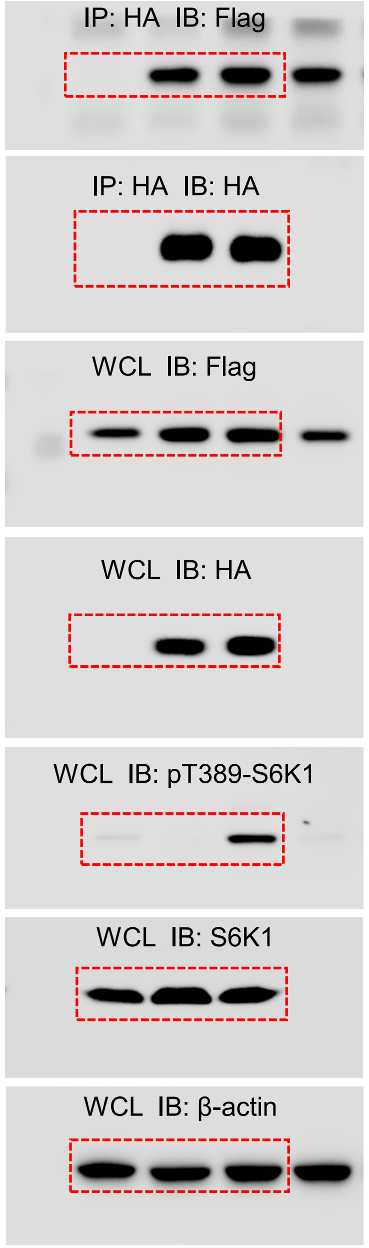

Full unedited blot for
Supplemental Figure 3C

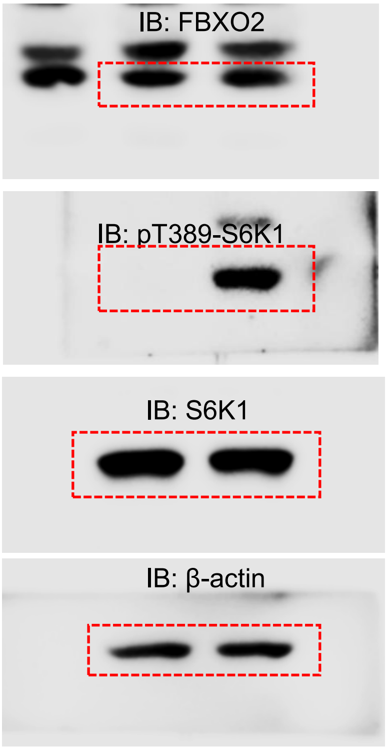

Full unedited blot for
Supplemental Figure 3F

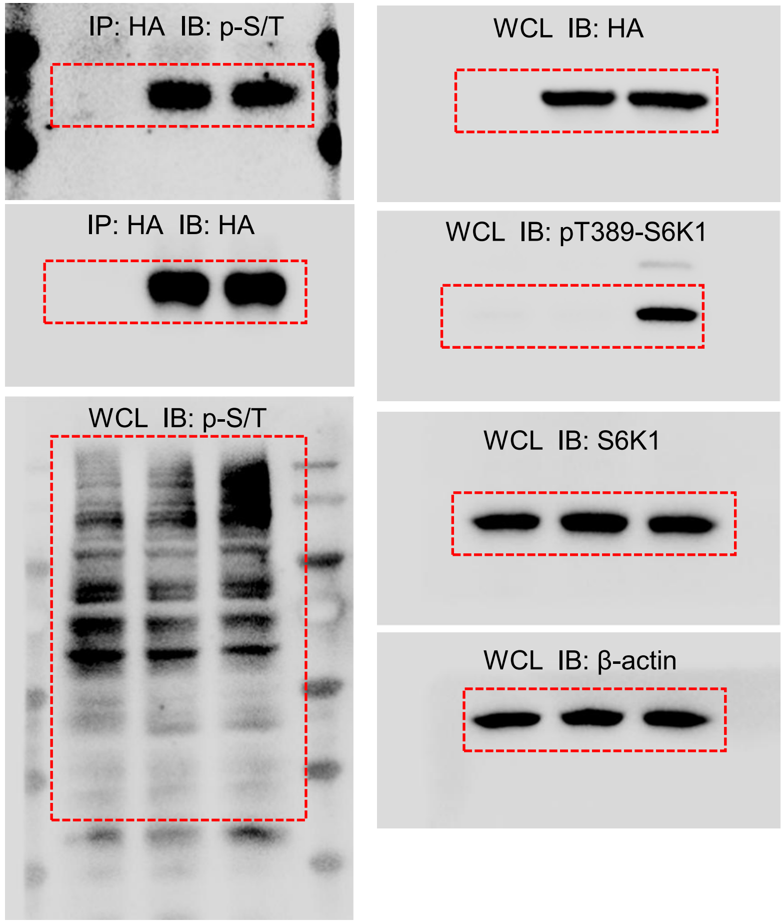

Full unedited blot for
Supplemental Figure 3G

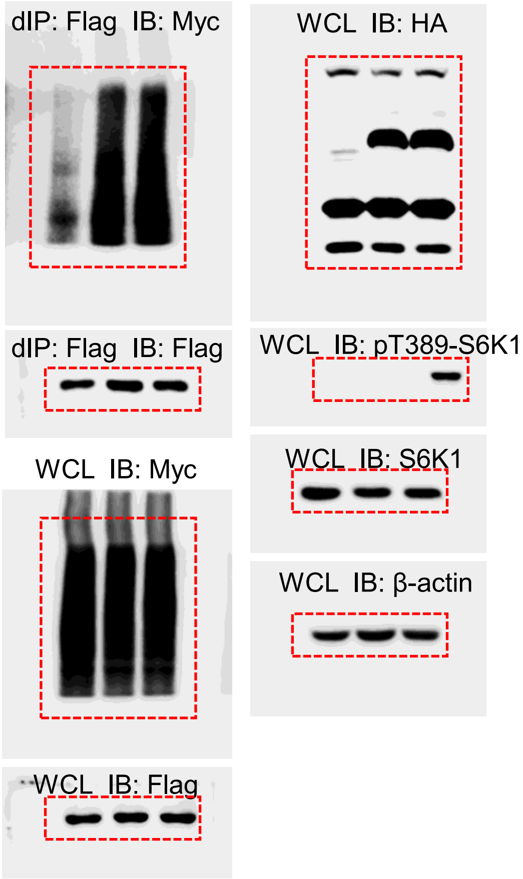

Full unedited blot for Supplemental Figure 4A

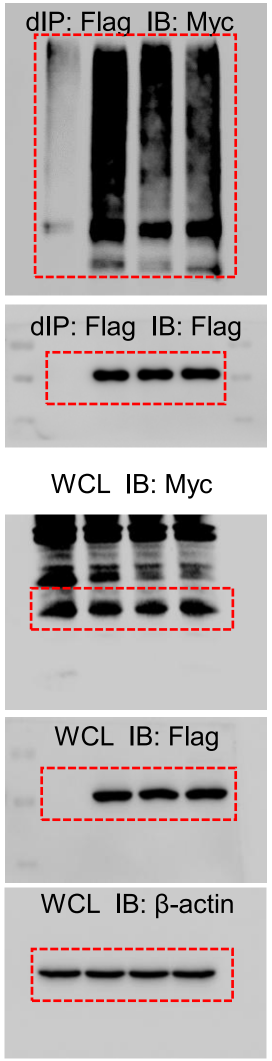

Full unedited blot for Supplemental Figure 4B

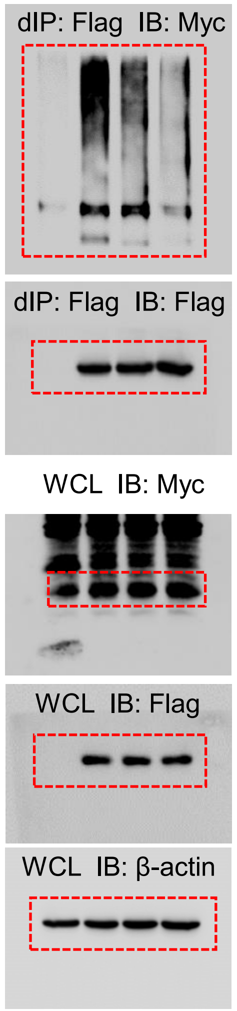

Full unedited blot for Supplemental Figure 4D

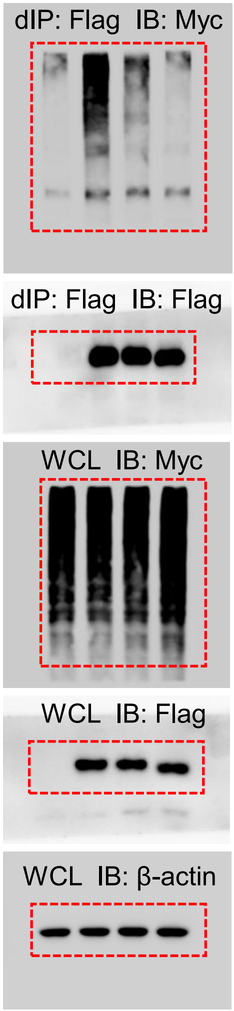

Full unedited blot for Supplemental Figure 4E

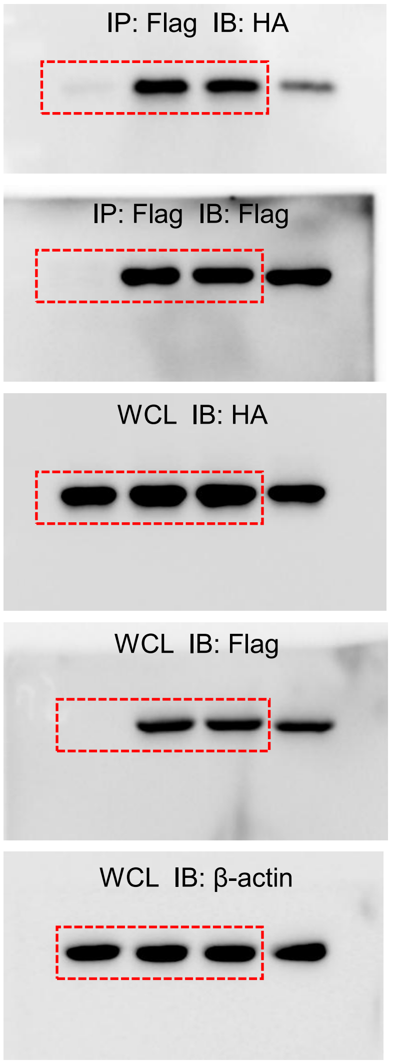

Full unedited blot for Supplemental Figure 5A

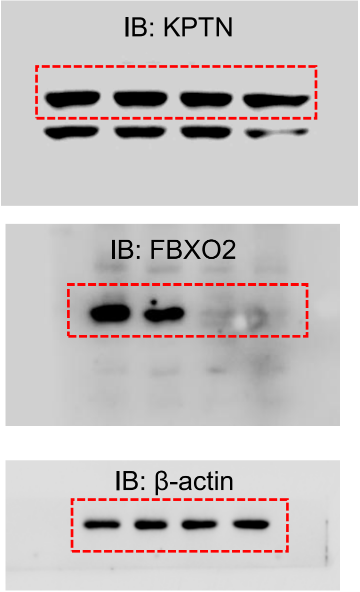

Full unedited blot for Supplemental Figure 5B

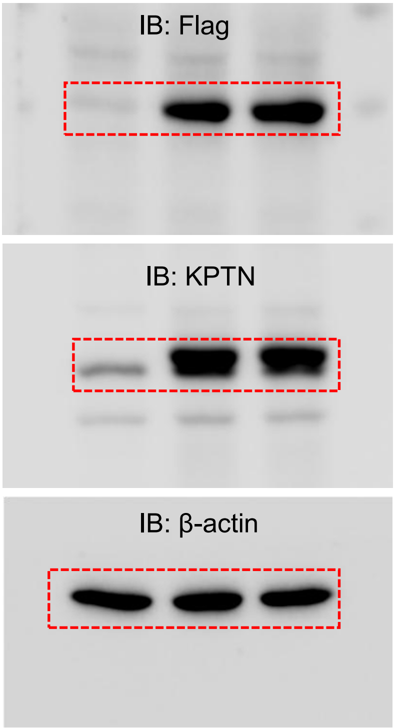

Full unedited blot for Supplemental Figure 5C

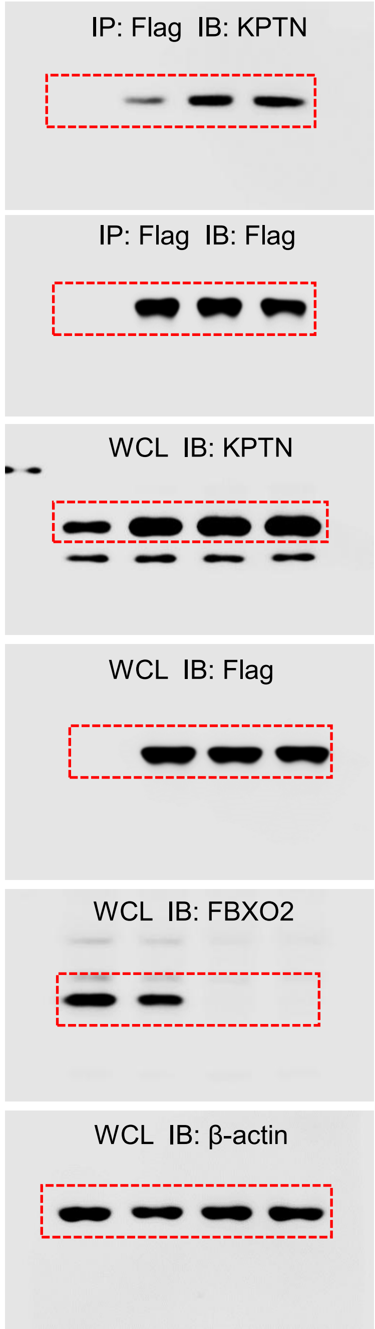

Full unedited blot for Supplemental Figure 5D

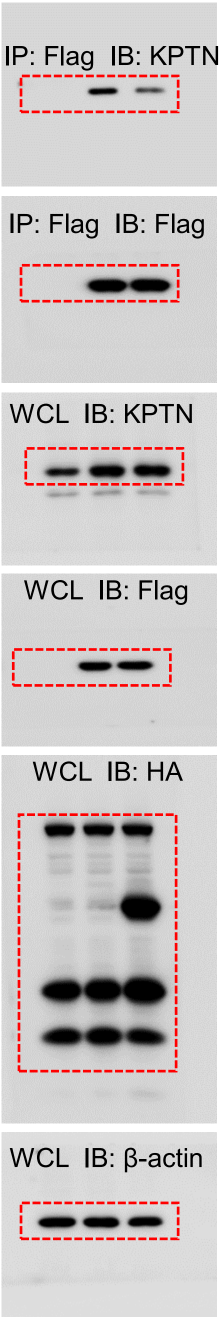

Full unedited blot for Supplemental Figure 5E

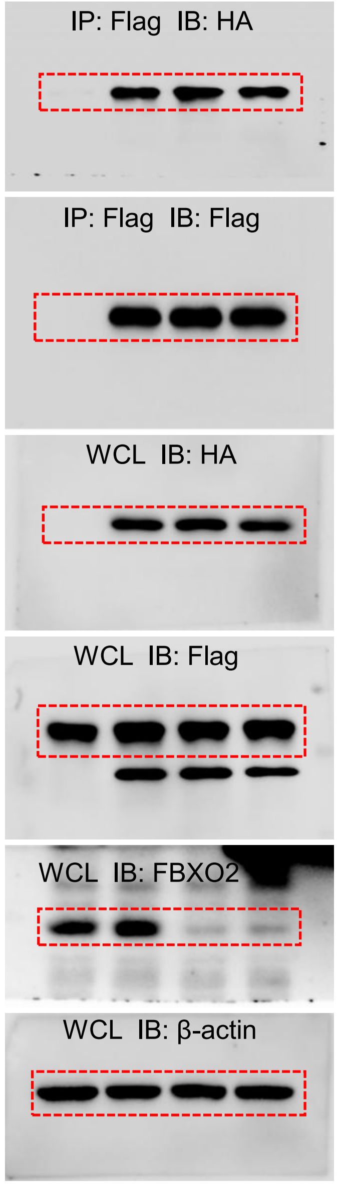

Full unedited blot for Supplemental Figure 5F

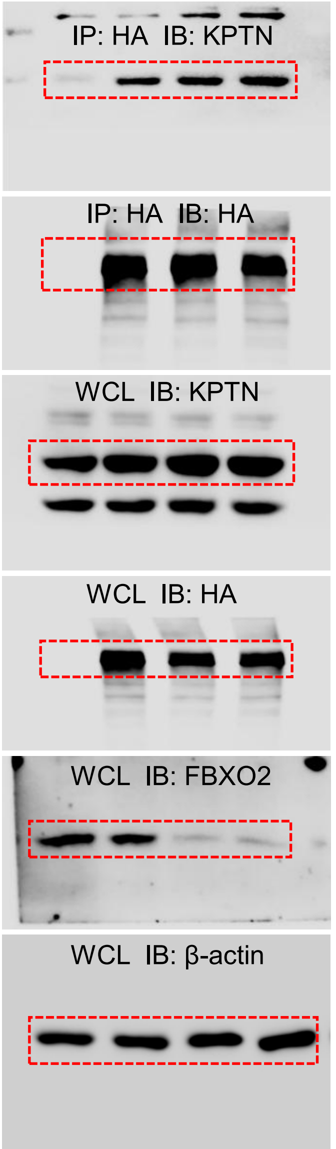

Full unedited blot for Supplemental Figure 5G

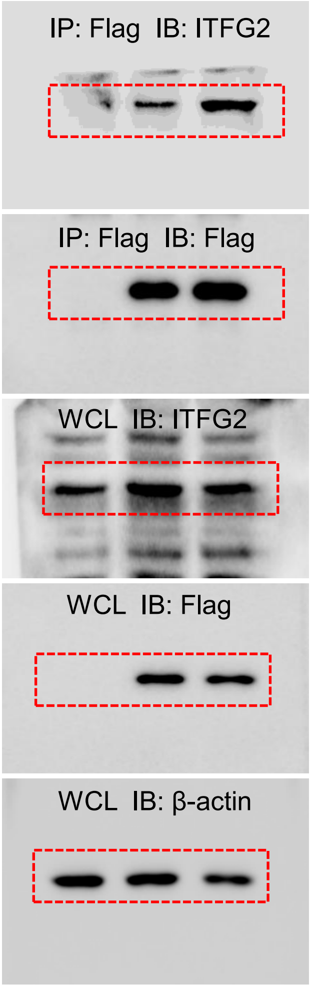

Full unedited blot for Supplemental Figure 5H

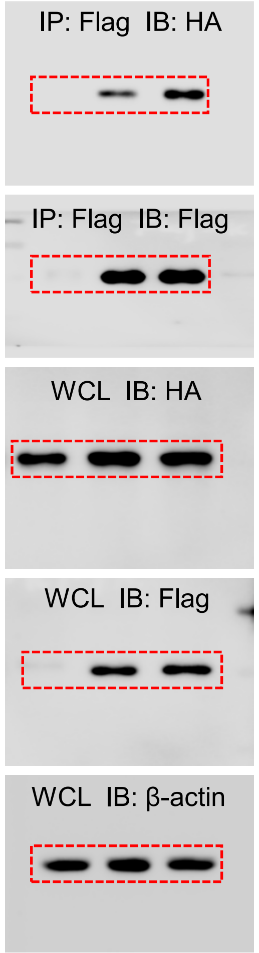

Full unedited blot for Supplemental Figure 5I

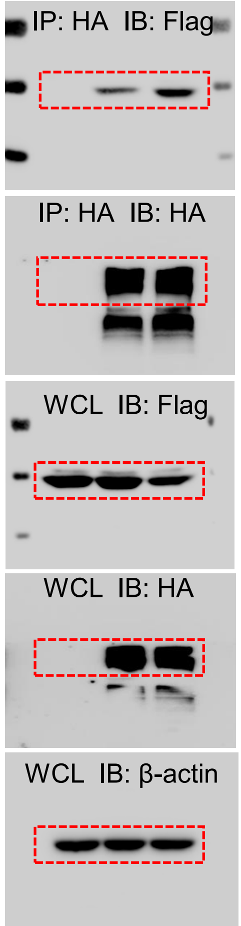

Supplement: Unedited blot and gel images [file jci-136-195031-s057.pdf]
